# Targeting acetylcholine signaling modulates persistent drug tolerance in EGFR-mutant lung cancer and impedes tumor relapse

**DOI:** 10.1172/JCI160152

**Published:** 2022-10-17

**Authors:** Meng Nie, Na Chen, Huanhuan Pang, Tao Jiang, Wei Jiang, Panwen Tian, LiAng Yao, Yangzi Chen, Ralph J. DeBerardinis, Weimin Li, Qitao Yu, Caicun Zhou, Zeping Hu

**Affiliations:** 1 School of Pharmaceutical Sciences, Tsinghua-Peking Center for Life Sciences, Beijing Frontier Research Center for Biological Structure, Tsinghua University, Beijing, China.; 2 Department of Medical Oncology, Shanghai Pulmonary Hospital, Tongji University Medical School Cancer Institute, Tongji University of Medicine, Shanghai, China.; 3 Affiliated Tumor Hospital of Guangxi Medical University, Nanning, Guangxi, China.; 4 Department of Respiratory and Critical Care Medicine, West China Hospital, Sichuan University, Chengdu, China.; 5 Children’s Medical Center Research Institute and; 6 Howard Hughes Medical Institute, University of Texas Southwestern Medical Center, Dallas, USA.

**Keywords:** Metabolism, Oncology, Drug therapy, Lung cancer, Tolerance

## Abstract

Although first-line epidermal growth factor receptor (EGFR) tyrosine kinase inhibitor (TKI) therapy is effective for treating EGFR-mutant non–small cell lung cancer (NSCLC), it is now understood that drug-tolerant persister (DTP) cells escaping from initial treatment eventually drives drug resistance. Here, through integration of metabolomics and transcriptomics, we found that the neurotransmitter acetylcholine (ACh) was specifically accumulated in DTP cells, and demonstrated that treatment with EGFR-TKI heightened the expression of the rate-limiting enzyme choline acetyltransferase (ChAT) in ACh biosynthesis via YAP mediation. Genetic and pharmacological manipulation of ACh biosynthesis or ACh signaling could predictably regulate the extent of DTP formation in vitro and in vivo. Strikingly, pharmacologically targeting ACh/M3R signaling with an FDA-approved drug, darifenacin, retarded tumor relapse in vivo. Mechanistically, upregulated ACh metabolism mediated drug tolerance in part through activating WNT signaling via ACh muscarinic receptor 3 (M3R). Importantly, we showed that aberrant ACh metabolism in patients with NSCLC played a potential role in predicting EGFR-TKI response rate and progression-free survival. Our study therefore defines a therapeutic strategy — targeting the ACh/M3R/WNT axis — for manipulating EGFR TKI drug tolerance in the treatment of NSCLC.

## Graphical abstract

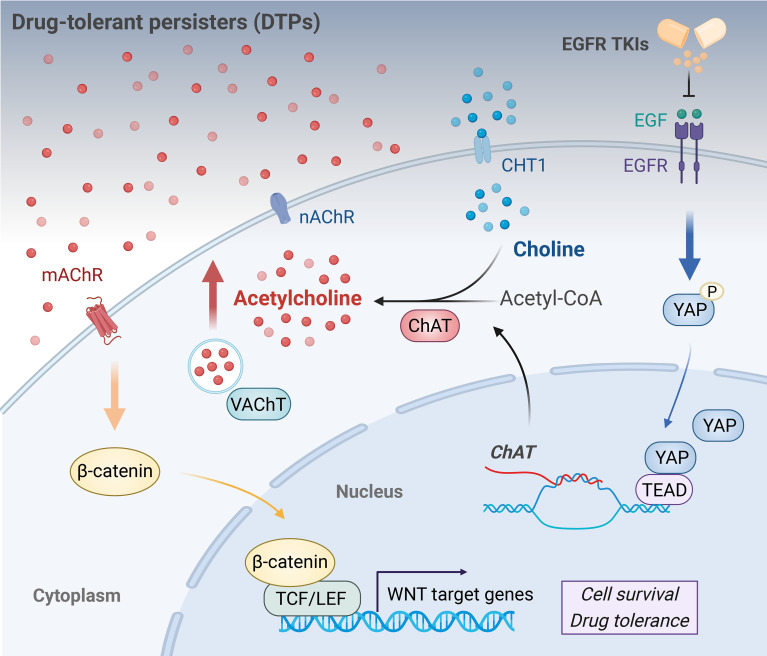

## Introduction

Patients with non–small cell lung cancer (NSCLC) with activating mutations in the epidermal growth factor receptor (EGFR) clearly benefit from EGFR–tyrosine kinase inhibitor (EGFR-TKI) therapy (1). However, like other targeted therapies, EGFR-TKIs lead to acquired resistance, and eventual tumor relapse compromises overall patient survival, highlighting the urgent need to develop new strategies for circumventing drug resistance (2, 3). Although many studies have been focused on the genetic mechanisms of drug resistance, emerging evidence suggests the importance of residual drug-tolerant persister (DTP) cancer cells, which survive the initial targeted therapy via diverse, reversible, and nonmutational mechanisms, such as transcriptional, epigenetic, and metabolic reprogramming (4–11). Recent studies have demonstrated an embryonic diapause–like adaption enabling the persistence under cancer treatment (12, 13). DTP cells, which underlie the phenomenon known as minimal residual disease (MRD), constitute a reservoir of slow-cycling cells that can drive irreversible acquired drug resistance upon long-term drug treatment, which eventually leads to cancer relapse (14–17). Accordingly, targeting DTP cells might provide a promising strategy to impede drug resistance and prolong treatment responses.

Metabolic reprogramming has been recently reported to contribute to both drug efficacy and to acquired drug resistance in cancer treatment, so targeting aberrantly regulated metabolic pathways, such as methionine metabolism and lipid metabolism, emerged as an attractive strategy for developing novel therapies (18–21). The fact that many reported therapy-induced, drug-tolerant states are transitory (and drug sensitivity reversible upon treatment withdrawal) clearly suggests roles for epigenetic or metabolic reprogramming (16). Mounting evidence has highlighted the functional impacts of epigenetic reprogramming on drug tolerance, including dysregulation of histone methylation (4, 22, 23). In contrast to the extensive studies that have explored epigenetic mechanisms, systematic investigation on metabolic reprogramming related to drug tolerance is rare.

Previous studies have revealed the contribution of neurotransmitter signaling to cancer initiation, progression, and metastasis (24–30). It has been recognized for some time that neurotransmitters can be released by cells outside the autonomic nervous system, for example by cancer cells and immune cells, and we know that neurotransmitters signal through their receptors on target cells in an autocrine or paracrine way (31–33). Notably, small molecules targeting neurotransmitter receptors have shown clinical potential for treating cancer (34–36). However, little evidence indicates the link between neurotransmitter signaling and drug tolerance.

Here, we initially detected an increase in levels of the neurotransmitter acetylcholine (ACh) in DTP cells. Extending these observations beyond in vitro work, we deployed an EGFR-TKI–regressed mouse model and also found the accumulation of ACh. Plausibly explaining the elevated ACh levels that we detected in both cell lines and mice, we established that administration of EGFR-TKI caused upregulated expression of the ACh biosynthesis enzyme choline acetyltransferase (ChAT) through YAP mediation. Genetic manipulation and pharmacological inhibition of ACh metabolism and signaling reduced DTP formation in vitro and in vivo. Furthermore, we demonstrated ACh-mediated drug tolerance in part through WNT signaling in an ACh muscarinic receptor 3–dependent (M3R-dependent) manner, constituting the ACh/M3R/WNT axis. Importantly, plasma ACh levels and tumor ChAT expression of human patients with NSCLC correlated with response to EGFR-TKI treatment and progression-free survival. Therapeutically, combination of EGFR-TKI with ACh/M3R signaling inhibition using the FDA-approved drug darifenacin impeded tumor relapse in mice, demonstrating a prospective combination therapeutic strategy to manipulate the emergence of drug tolerance.

## Results

### The neurotransmitter ACh accumulates in an EGFR-TKI–induced tolerant state in vitro and in vivo.

**Figure 1:**
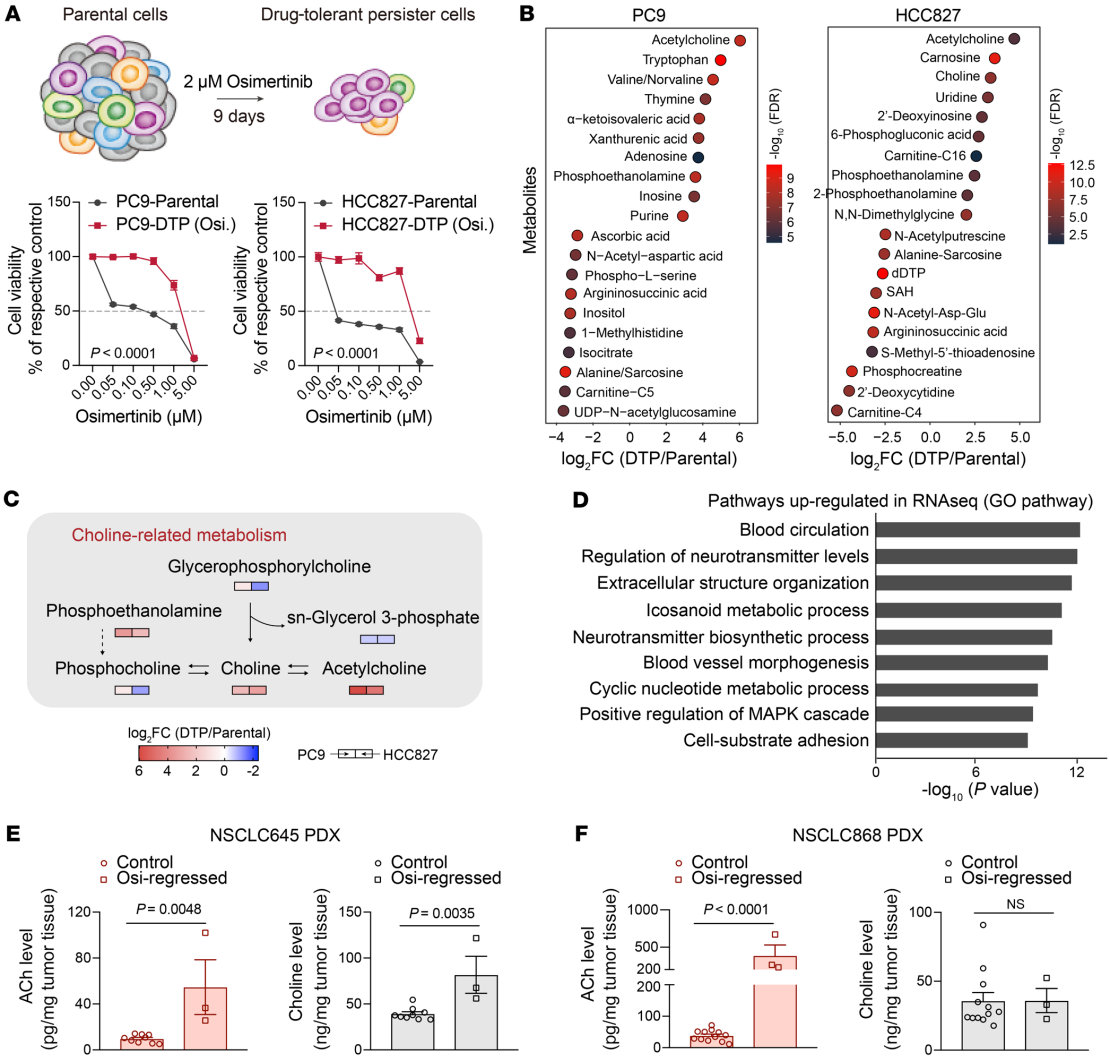
Accumulation of neurotransmitter ACh in EGFR-TKI–induced DTP cells and regressed tumors from NSCLC mouse xenografts. (**A**) Top: Schematic representation of the establishment of DTP cells. Bottom: Dose-response curves of parental and DTP cells incubated in increasing concentrations of osimertinib for 72 hours (*n* = 5). (**B**) Metabolites differentiating between parental and DTP cells (FDR < 0.05, fold change > 1.5 or < 0.67). Top 10 upregulated or downregulated metabolites with absolute log_2_(fold change) > 2 are shown. (**C**) Changes in metabolites associated with choline metabolism in PC9-derived and HCC827-derived DTP cells relative to parental PC9 and HCC827 cells. Log_2_(fold change) (DTP cells/parental cells) of metabolites are represented by color intensity. (**D**) GO analysis of upregulated genes in PC9-derived DTP cells compared with parental PC9 cells. (**E**) Quantification of ACh and choline levels in control and osimertinib-regressed tumors derived from NSCLC645 mouse PDX models. *n* = 3 mice; 3 fragments were harvested for analysis in each tumor of controls. (**F**) Quantification of ACh and choline levels in control and osimertinib-regressed tumors derived from NSCLC868 mouse PDX models. *n* = 3 or 4 mice; 3 fragments were harvested for analysis in each tumor of controls. In **A**, **E**, and **F**, data are shown as mean ± SEM. Significance was assessed using 2-way ANOVA adjusted by Bonferroni’s correction (**A**) or 2-tailed Student’s *t* test (**E** and **F**).

Our initial in vitro experimental model consisted of *EGFR*-mutant human NSCLC cell lines PC9 and HCC827, which we exposed to a lethal dose of EGFR-TKIs for 9 days (gefitinib or osimertinib); this treatment resulted in the expected small population of viable and quiescent DTP cells. Notably, compared with parental cells, the DTP cells exhibited reduced sensitivity to EGFR-TKIs and entered a nonmutationally slow-cycling state (Figure 1A and Supplemental Figure 1, A–J; supplemental material available online with this article; https://doi.org/10.1172/JCI160152DS1). To identify potential metabolic vulnerabilities in DTP cells, we performed a liquid chromatography and mass spectrometry (LC-MS) targeted metabolomics experiment comparing PC9 and HCC827 cells alongside DTPs respectively derived from these NSCLC cell lines (Supplemental Figure 2, A–D). Principal component analysis indicated that the DTP cells were metabolically distinct from parental cells (Supplemental Figure 2, E and F). Interestingly, we found that the abundance of several metabolites from choline metabolism were significantly altered in PC9- and HCC827-derived DTP cells. Strikingly, the ACh, widely known as a neurotransmitter, was significantly accumulated in both PC9-derived and HCC827-derived DTP cells compared with parental cells (Figure 1, B and C). We next performed RNA sequencing (RNA-seq) of parental PC9 cells and PC9-derived DTP cells. Gene Ontology (GO) analysis of RNA-seq data also correlated DTP cells with the elevated expression of the genes involved in regulating neurotransmitter levels, especially for their biosynthesis (Figure 1D). Collectively, for 2 independent cell lines and their derived DTP cells, it appears that EGFR-TKI treatment induces reprogramming of ACh metabolism.

To determine whether the accumulation of ACh also occurs in EGFR-TKI–regressed tumors, we investigated the changes in ACh metabolism in osimertinib-regressed patient-derived xenograft (PDX) tumors (37) established from *EGFR*-mutant NSCLC patients. Specifically, for these PDX mice, osimertinib treatment rapidly suppressed and stabilized the tumor volume (Supplemental Figure 3, A and B). The osimertinib-regressed tumors exhibited desmoplasia and cell pleomorphism with aberrant nuclear and cytoplasmic shape (Supplemental Figure 3, C and D), consistent with the observations in previous studies (12, 15). Then, we performed metabolomics analysis of the PDXs, which also revealed a distinct metabolic profile of osimertinib-regressed tumors compared with control tumors and altered metabolites associated with choline metabolism (Supplemental Figure 4, A–D). Moreover, quantitative measurement of ACh in these PDXs showed an elevated production of ACh in osimertinib-regressed tumors (Figure 1, E and F).

**Figure 2:**
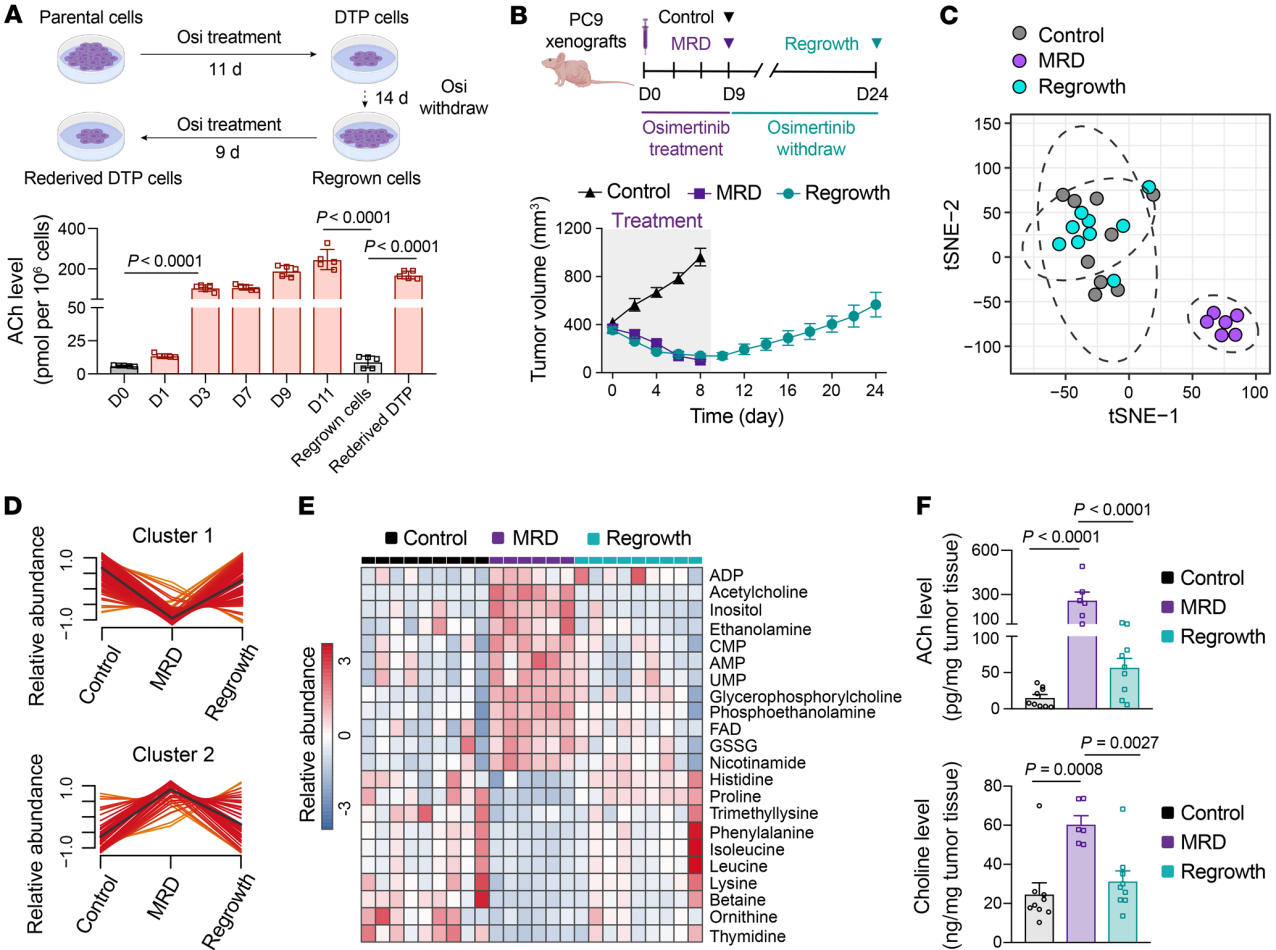
The reversible ACh levels and metabolic profiles of drug tolerance state in vivo. (**A**) Quantification of ACh levels in PC9 cells treated with 2 μM osimertinib for various time periods (*n* = 5). (**B**) PC9-xenograft mice were treated with 5 mg/kg osimertinib alone for 9 days and then osimertinib was withdrawn. Tissue samples were collected from control (*n* = 9), MRD (*n* = 6), and regrown (*n* = 9) tumors. (**C**) t-SNE plot of control (*n* = 9), MRD (*n* = 6), and regrown (*n* = 9) tumors according to metabolites detected from targeted metabolomics. (**D**) Clustering of metabolites detected in control (*n* = 9), MRD (*n* = 6), and regrown (*n* = 9) tumors. Black lines represent the average trajectory for each cluster. (**E**) Heatmap showing the abundance of differential metabolites (FDR < 0.05, fold change > 1.5 or < 0.67) with reversibility among control, MRD, and regrown tumors. Color intensity represents metabolite abundance. (**F**) Quantification of ACh and choline levels in control (*n* = 9), MRD (*n* = 6), and regrown tumors (*n* = 9) derived from PC9-xenograft model. In **A** and **F**, data are shown as mean ± SEM. Significance was assessed using 1-way ANOVA with Tukey’s test.

Next, we investigated whether ACh metabolism reprogramming is transient and reversibly associated with a drug-tolerant cell state. These experiments comprise 3 phases: initial culturing of PC9 cells with osimertinib, withdrawal of osimertinib, and redosing with osimertinib. Cells regained sensitivity to osimertinib upon withdrawal of osimertinib (Supplemental Figure 5A). Redosing with osimertinib also led to the reestablishment of small population of rederived DTP cells, a finding which demonstrates metabolic reversibility (i.e., sensitivity to osimertinib) rather than genetic mutations for the DTP state (Supplemental Figure 5A). EGFR-TKI treatment led to a time-dependent accumulation of ACh levels in cells, and importantly, monitoring of ACh levels also revealed dynamically fluctuating ACh levels that corresponded to the sequential drug-tolerant or drug-sensitive states in the 3 phases; ACh levels were high in the initial DTP cells and in the rederived DTP cells that developed upon redosing, whereas the parental cells and the regrown cells from the osimertinib withdrawal phase had relatively low ACh levels (Figure 2A).

In vivo experiments were performed with PC9 xenografts in which EGFR-TKI treatment was given to establish osimertinib-regressed MRD tumors by treating mice daily with either vehicle or a high dose of osimertinib (5 mg/kg) for 9 consecutive days, and followed by drug withdrawal to develop regrown tumors (Figure 2B). Interestingly, metabolomics analysis showed that the metabolic profiles in regrown tumors closely resembled those in control tumors, which were distinct from MRD tumors (Figure 2C and Supplemental Figure 5B). Through performing c-means clustering analysis of the detected metabolites, we found that several metabolites showed reversible changes, exhibiting a specific increase or decrease in MRD tumors, including metabolites associated with choline metabolism. As expected, MRD tumors had higher levels of ACh compared with regrown tumors or control tumors (Figure 2, D and E, and Supplemental Figure 5C). Moreover, quantitative measurement of ACh showed significantly increased levels of ACh and choline in MRD tumors of PC9-xenograft mice as compared with controls or regrown tumors (Figure 2F). These results are consistent with our in vitro observations of PC9 cells, supporting the idea that ACh levels are indeed transiently elevated in a residual drug-tolerant state upon EGFR-TKI treatment.

### EGFR-TKI treatment induces the increased expression of ChAT.

**Figure 3:**
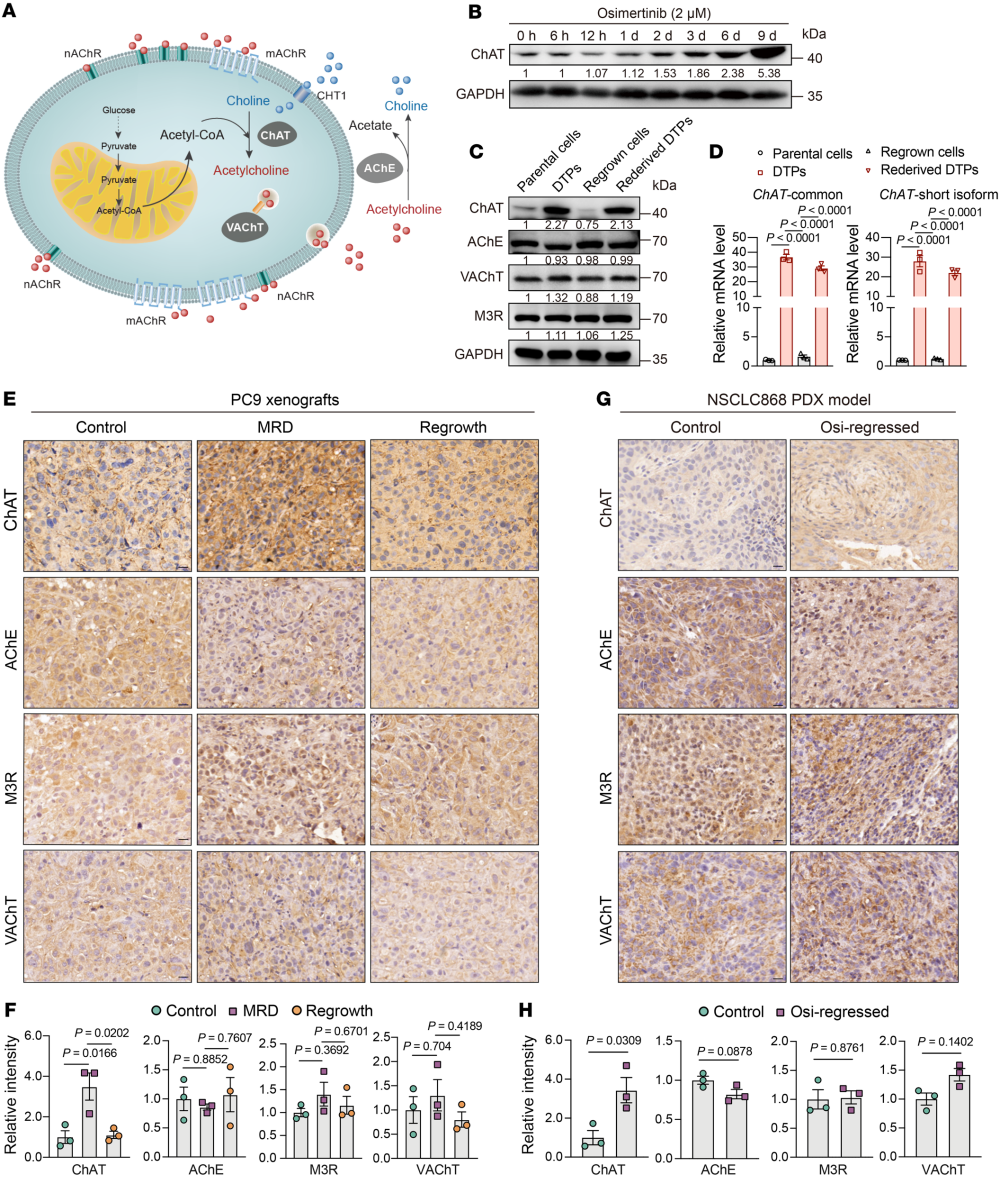
EGFR-TKI treatment heightens the expression of ChAT. (**A**) Schematic of the ACh biosynthesis, secretion, signaling activation, and degradation. (**B**) Relative protein levels of the short ChAT isoform in PC9 cells treated with 2 μM osimertinib for various time periods. (**C**) Relative protein levels of ACh metabolism modulators in parental PC9 cells, DTPs, regrown cells, and rederived DTPs. (**D**) Relative *ChAT* mRNA levels in parental PC9 cells, DTPs, regrown cells, and rederived DTPs analyzed by qPCR with primers for common and short isoform–specific exons of *ChAT* (*n* = 3). (**E** and **F**) Representative image and quantification of immunohistochemical staining of ChAT, VAChT, M3R, and AChE on the indicated control, MRD, and regrown tumor sections from PC9 xenografts (*n* = 3 mice per group). Scale bars: 20 μm. (**G** and **H**) Representative image and quantification of immunohistochemical staining of ChAT, VAChT, M3R, and AChE on the indicated control and osimertinib-regressed tumor sections from the PDX model (*n* = 3 mice per group). Scale bars: 20 μm. In **D**, **F**, and **H**, data are shown as mean ± SEM. Significance was assessed using 1-way ANOVA with Tukey’s test (**D** and **F**) or 2-tailed Student’s *t* test (**H**).

Having detected and confirmed the upregulation of ACh biosynthesis in DTP cells both in vitro and in vivo, we next investigated whether the observed metabolic differences were accompanied by corresponding genetic changes in the pathway. ACh is synthesized from choline and acetyl-CoA by the rate-limiting enzyme ChAT, and translocated into synaptic vesicles by the vesicular acetylcholine transporter (VAChT). After being secreted into the extracellular environment, ACh can engage nicotinic ACh receptors (nAChRs) and muscarinic ACh receptors (mAChRs) on target cells, and then degraded by acetylcholine esterase (AChE) (Figure 3A). To elucidate the possible causes of ACh accumulation in DTP cells, we assessed the protein expression of components associated with ACh metabolism and signaling. Notably, we observed upregulated expression of one short ChAT isoform (41 kDa), which has been previously identified in human bronchial epithelial cells (38), and a slight increase in VAChT and negligible alteration in other components like AChE, M3R, or choline transporter 1 (CHT1) (Supplemental Figure 6, A and B). In fact, it has been known that the gene encoding ChAT contains multiple exons, which undergo alternative splicing to generate variable transcripts (39, 40). To thoroughly investigate the alternative splicing of the *ChAT* gene, we explored the Ensembl and Human Protein Atlas databases, and found that the *ChAT* gene had a total of 12 splice variants, including 6 protein-coding variants. Of note, 1 splice variant, ChAT-212 (Ensembl database), contained 369 amino acids and could be translated into a short ChAT isoform with a molecular weight of 41 kDa (Supplemental Figure 6C). Alternative splicing is tightly modulated in different tissues, cell types, differentiation stages, and diseases (41, 42). Compared with the mouse brain and human normal lung fibroblast cells, we observed that the short ChAT isoform (41 kDa) was expressed in *EGFR*-mutant NSCLC cell lines at both mRNA and protein levels (Supplemental Figure 6, D and E). Moreover, we also found the expression of short ChAT isoform at mRNA and protein levels in tumor and adjacent normal lung tissues derived from NSCLC patients with or without *EGFR* mutation (Supplemental Figure 6, F and G).

Interestingly, in line with the observation that EGFR-TKI treatment led to a time-dependent accumulation of ACh levels in cells, we also found the time-dependent upregulation of short ChAT isoform expression (Figure 3B). Notably, the reversibly dynamic changes similar to ACh levels were also evident when we examined the short ChAT isoform at both mRNA and protein levels (Figure 3, C and D). Immunohistochemical analysis showed that the highest detected ChAT expression, a slight increase in VAChT, and no change in AChE and M3R were in MRD tumors compared with the control and/or regrown tumors in the PC9 xenografts (Figure 3, E and F). Additionally, we also observed reversible changes in p-EGFR, p-AKT, and p-ERK expression along with osimertinib treatment or withdrawal (Supplemental Figure 7A). Moreover, the analysis of PDX models also revealed a massive upregulated expression of ChAT, a slight increase in VAChT, and no change in AChE and M3R in osimertinib-regressed tumors (Figure 3, G and H). Notably, the analysis with The Cancer Genome Atlas (TCGA) database to investigate the clinical relevance of ChAT expression in *EGFR*-mutant NSCLC patients showed that patients with high ChAT levels were associated with a reduction in overall survival (Supplemental Figure 7B), suggesting there may exist an intrinsic aggressiveness in tumor cells expressing high levels of ChAT.

**Figure 4:**
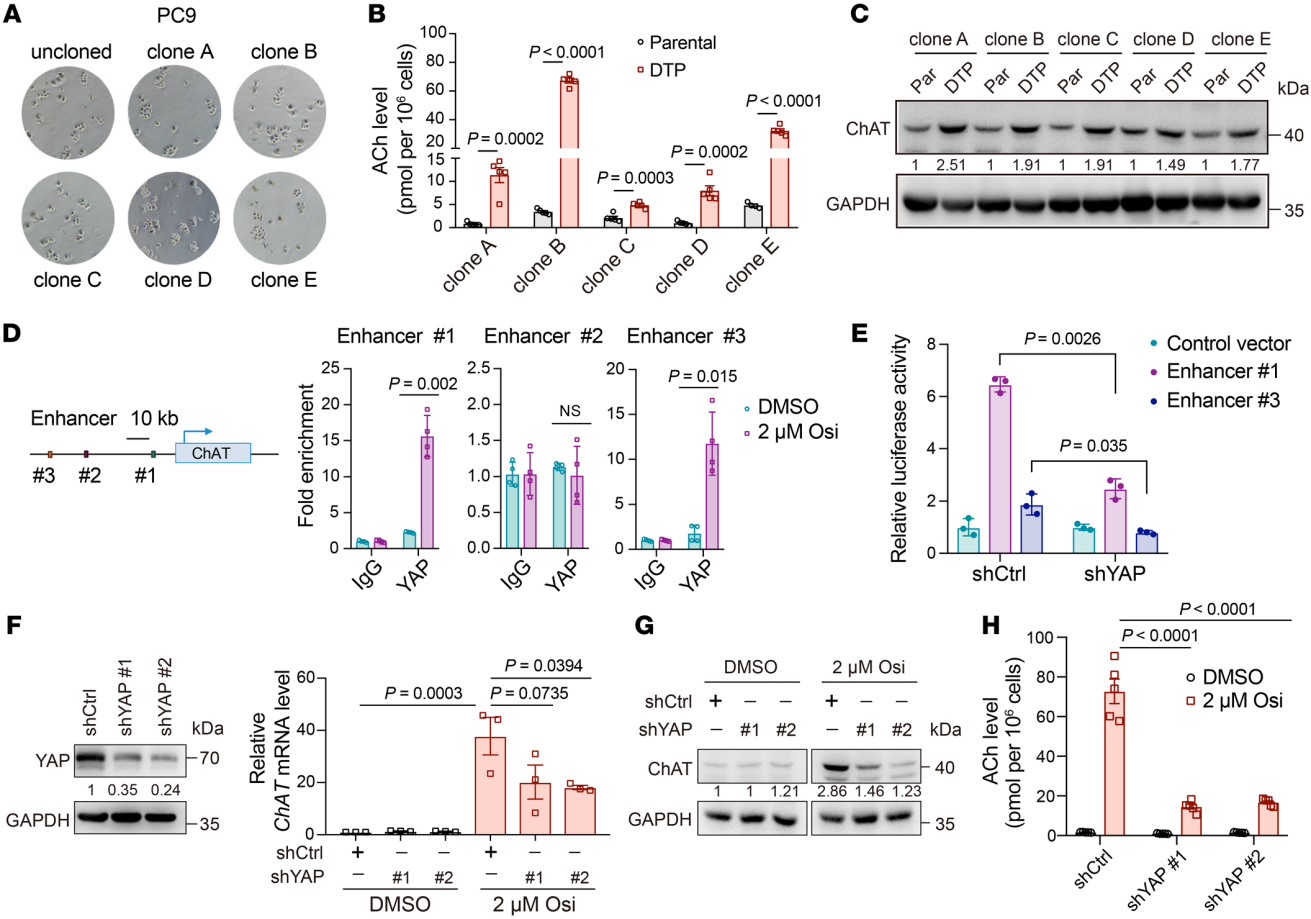
ChAT expression is activated transcriptionally through YAP mediation. (**A**) Microscopic image showing the uncloned PC9 cells or cells from 5 different single-cell-derived PC9 clones treated with 2 μM osimertinib for 9 days to identify DTPs. (**B**) Quantification of ACh levels in 5 different single-cell-derived PC9 clones treated with 2 μM osimertinib for 9 days (*n* = 5). (**C**) Protein levels of the short ChAT isoform in 5 different single-cell-derived PC9 clones treated with 2 μM osimertinib for 9 days. (**D**) ChIP-qPCR analyses in PC9 cells showing YAP bound to the enhancers 1 and 3 of the *ChAT* gene after 2 μM osimertinib treatment for 48 hours (*n* = 4). (**E**) Luciferase reporter assay in PC9 cells with stable silencing of YAP or control shRNA (shCtrl) treated with 2 μM osimertinib for 72 hours (*n* = 3). (**F**) Relative *ChAT* mRNA levels in PC9 cells with shYAP or shCtrl treated with 2 μM osimertinib for 9 days. Gene silencing was confirmed by Western blot on the left. (**G**) Relative protein levels of the short ChAT isoform in PC9 cells with shYAP or shCtrl treated with 2 μM osimertinib for 9 days. (**H**) Quantification of ACh levels in PC9 cells with shYAP or shCtrl treated with 2 μM osimertinib for 9 days (*n* = 5). In **B**, **D**–**F**, and **H**, data are shown as mean ± SEM. Significance was assessed using 2-way ANOVA with Tukey’s test (**B**, **D**, **E**, and **H**) or 1-way ANOVA with Tukey’s test (**F**).

Next, we were prompted to determine whether the ACh/ChAT increase is an adaptive response to EGFR-TKI or the result of a positive selection of cells with high expression of the short ChAT isoform. We isolated PC9 single-cell clones and found that all single-cell-derived PC9 clones could yield DTPs (Figure 4A), which is consistent with a previous report (4). Importantly, DTPs derived from clonal PC9 cells showed robust accumulation of ACh to varying degrees (Figure 4B), possibly due to heterogeneity within the population. In accordance with the increased ACh levels, we also found upregulated expression of the short ChAT isoform in each single-cell-derived PC9 clone upon osimertinib treatment (Figure 4C). Taken together, these findings demonstrated that the ACh/ChAT increase was, at least in part, an adaptive response upon EGFR-TKI treatment, although we could not exclude the possibility of intrinsically high levels of the short ChAT isoform in tumor cells. Further, we investigated the mechanism by which osimertinib upregulated short ChAT isoform expression. Because we have clearly shown that osimertinib exposure reversibly modulated the mRNA and protein levels of ChAT, we hypothesized that osimertinib-induced upregulation of ChAT may be mediated through transcriptional regulation.

A previous study has demonstrated the necessity of YAP activation for evading combined EGFR/MEK inhibition in cancer cells (9). We analyzed the public chromatin immunoprecipitation–sequencing (ChIP-seq) data from the previous studies related to drug tolerance and multiple human cell lines, which were integrated by ChIP-Atlas (9), finding 3 potential YAP-binding sites in the distal regulatory regions of *ChAT* and increased ChIP-seq signal in these regions upon drug treatment. In particular, an increased H3K27ac ChIP-seq signal was observed in the regions overlapping the YAP-binding sites, which has been found to be an active enhancer mark (43, 44) (Supplemental Figure 8A). Hence, these observations prompted us to speculate that YAP may modulate *ChAT* transcription through binding to the distal enhancers. Indeed, ChIP-qPCR revealed that osimertinib treatment significantly led to increased YAP binding on enhancers 1 and 3 of *ChAT* and no change in YAP binding on enhancer 2 (Figure 4D). Consistently, luciferase reporter assays demonstrated that YAP knockdown resulted in a significant reduction in the reporter activity of enhancers 1 and 3 under osimertinib treatment (Figure 4E). To further confirm this, we stably silenced YAP with shRNA in PC9 cells and found that YAP knockdown reduced the osimertinib-induced upregulation of the short ChAT isoform at both mRNA and protein levels (Figure 4, F and G). In addition, a YAP/TEAD inhibitor, verteporfin, could also inhibit the upregulation of mRNA and protein levels of the short ChAT isoform induced by osimertinib, indicating that the regulatory role of YAP was dependent on the YAP/TEAD interaction (Supplemental Figure 8, B and C). Importantly, osimertinib-induced accumulation of ACh was also attenuated by YAP knockdown (Figure 4H). Taken together, these findings demonstrate that *ChAT* is a direct transcriptional target of YAP through mediating distal enhancer sites.

### Aberrant ACh metabolism and signaling is necessary for drug tolerance formation in vitro and in vivo.

**Figure 5:**
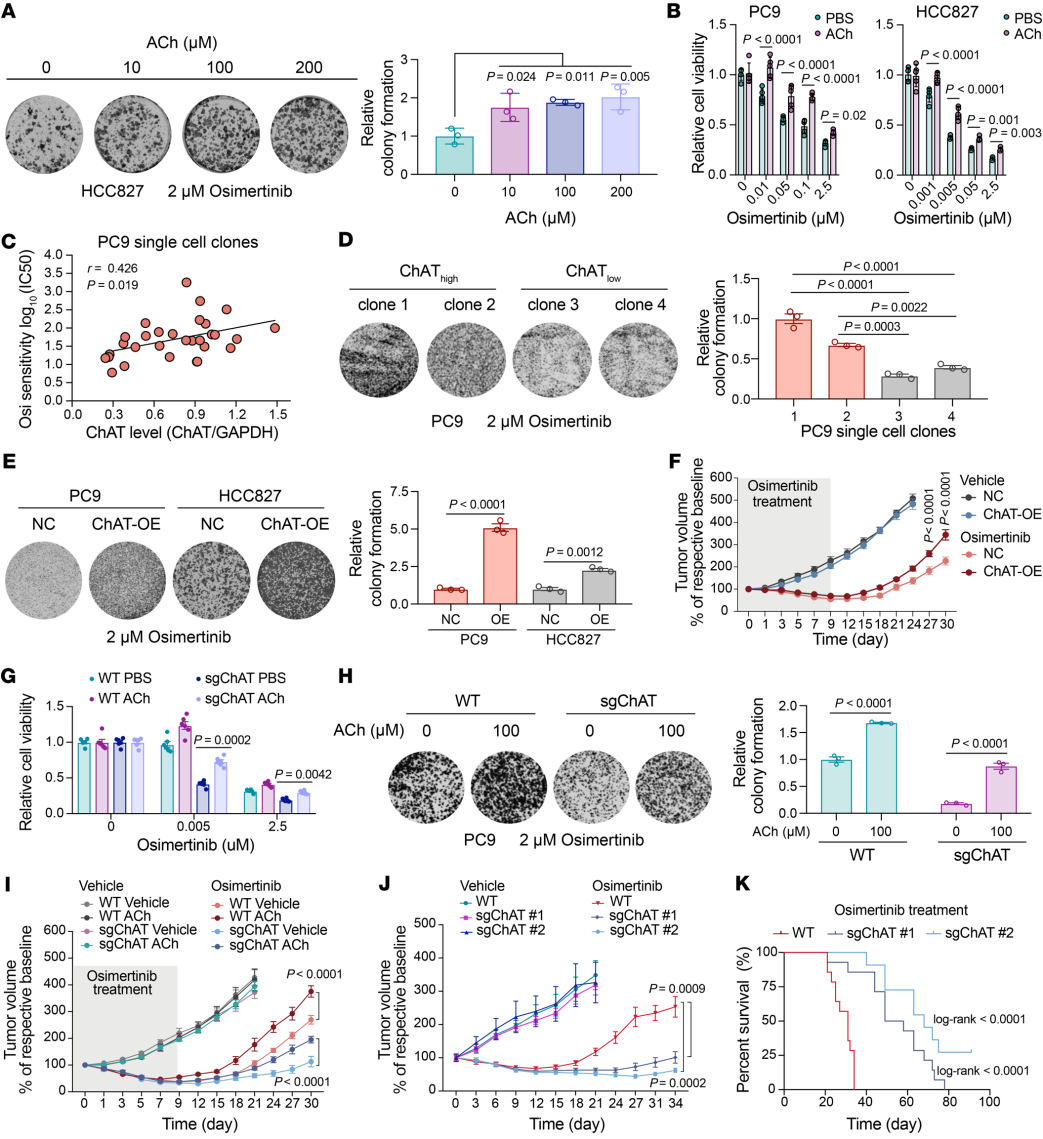
Activated ACh metabolism and signaling promotes tolerance to EGFR inhibition in vitro and in vivo. (**A**) Colony formation assay of cells treated with indicated drugs (*n* = 3). (**B**) Relative viability of cells treated with osimertinib and 10 μM ACh (*n* = 5). (**C**) Linear regression analysis of correlation between short ChAT isoform levels and osimertinib sensitivity log_10_(IC_50_). (**D**) Colony formation assay of PC9 single-cell clones exposed to osimertinib (*n* = 3). (**E**) Colony formation assay of Flag-ChAT short isoform overexpression (ChAT-OE, 41 kDa) and negative control (NC) cells treated with osimertinib (*n* = 3). (**F**) PC9-xenograft mice injected with ChAT-OE or NC cells were treated with 5 mg/kg osimertinib for 9 days or with vehicle. *n* = 7 or 8 per group. (**G**) Relative viability of PC9 WT and *ChAT*-knockout cells treated with osimertinib and 10 μM ACh (*n* = 6). (**H**) Colony formation assay of PC9 WT and *ChAT*-knockout cells treated with osimertinib and ACh (*n* = 3). (**I**) PC9-xenograft mice injected with WT and *ChAT*-knockout cells were treated with 5 mg/kg osimertinib for 9 days or with vehicle. ACh was injected subcutaneously once daily (*n* = 7). (**J**) PC9-xenograft mice injected with WT and *ChAT*-knockout cells were treated with 1 mg/kg osimertinib or with vehicle. *n* = 5 or 6 per vehicle group, *n* = 11 or 14 per osimertinib treatment group. (**K**) Percentage survival curve generated from PC9-xenograft mice. In **A**, **B**, and **D**–**J**, data are shown as mean ± SEM. Significance was assessed using 1-way ANOVA with Dunnett’s test (**A**), 2-way ANOVA with Tukey’s test (**B** and **G**), 1-way ANOVA with Tukey’s test (**D**, **E** and **H**), 2-way ANOVA adjusted by Bonferroni’s correction (**F**, **I** and **J**), or 2-sided log-rank test (**K**).

Given our detection of reprogrammed ACh metabolism in DTP cells, we reasoned that it may be possible to modulate DTP formation by genetically and/or pharmacologically manipulating ACh accumulation, ACh secretion, or ACh signal reception. Firstly, we found that cotreatment of parental HCC827 cells with osimertinib plus exogenous ACh promoted the DTP formation (Figure 5A). In addition, exogenous ACh treatment impaired the drug sensitivity to osimertinib in PC9 and HCC827 cells (Figure 5B). To further clarify the function of the short ChAT isoform, we isolated PC9 single-cell clones, which exhibited heterogeneity in short ChAT isoform levels prior to drug treatment. Interestingly, a positive correlation between short ChAT isoform levels and osimertinib sensitivity (log__10__[IC__50__]) was observed (Figure 5C). We identified distinct ChAT^^hi^^ and ChAT^^lo^^ single-cell clones that were consistent with respective ACh levels and found that single-cell clones with high ChAT levels exhibited enhanced DTP formation compared with those with low ChAT levels upon osimertinib treatment (Supplemental Figure 9A and Figure 5D). Moreover, overexpression of the short ChAT isoform induced a significant increase in ACh levels and markedly increased the DTP formation (Supplemental Figure 9B and Figure 5E). Notably, to determine the functional role of the short ChAT isoform in vivo, we established the PC9 xenograft models and found that short ChAT isoform overexpression enhanced drug tolerance and further led to a more rapid tumor relapse (Figure 5F). In addition, we also overexpressed the full-length ChAT, which induced a marked increase in ACh levels, and observed that full-length ChAT overexpression enhanced DTP formation in vitro and accelerated tumor relapse in vivo (Supplemental Figure 9, C–F), suggesting that the increase in ACh induced by ChAT plays an important role in conferring drug tolerance.

Conversely, we assessed the effect of *ChAT* knockout on DTP cell formation using the CRISPR/Cas9 system. The osimertinib-induced ACh increase was significantly blocked with *ChAT* knockout (Supplemental Figure 9G). Notably, we found that *ChAT* knockout enhanced the sensitivity to osimertinib and this effect was rescued by supplementation of exogenous ACh (Figure 5G). Consistently, *ChAT* knockout markedly suppressed the DTP cell formation, which was also rescued by supplementation of exogenous ACh (Figure 5H). Importantly, to further confirm these observations in vivo, we used PC9 WT and *ChAT*-knockout cells pretreated with or without exogenous ACh for 3 days to produce xenograft tumors in nude mice. The result showed that *ChAT* knockout caused slow tumor relapse, and this effect was rescued through injection of exogenous ACh (Figure 5I). In addition, with long-term osimertinib treatment, *ChAT* knockout significantly enhanced drug response and inhibited tumor relapse, and improved survival outcome (Figure 5, J and K). The similar in vitro and in vivo observations in *ChAT*-knockout cells were also noted in cells in which *ChAT* was knocked down using siRNA and shRNA (Supplemental Figure 9, H–L). Notably, knockdown of M3R or VAChT by shRNA sensitized PC9 and HCC827 cells to osimertinib or gefitinib inhibition and decreased the extent of DTP cell formation (Supplemental Figure 10, A–D). Taken together, these findings indicate a potential functional link between altered ACh metabolism and signaling and EGFR inhibition.

**Figure 6:**
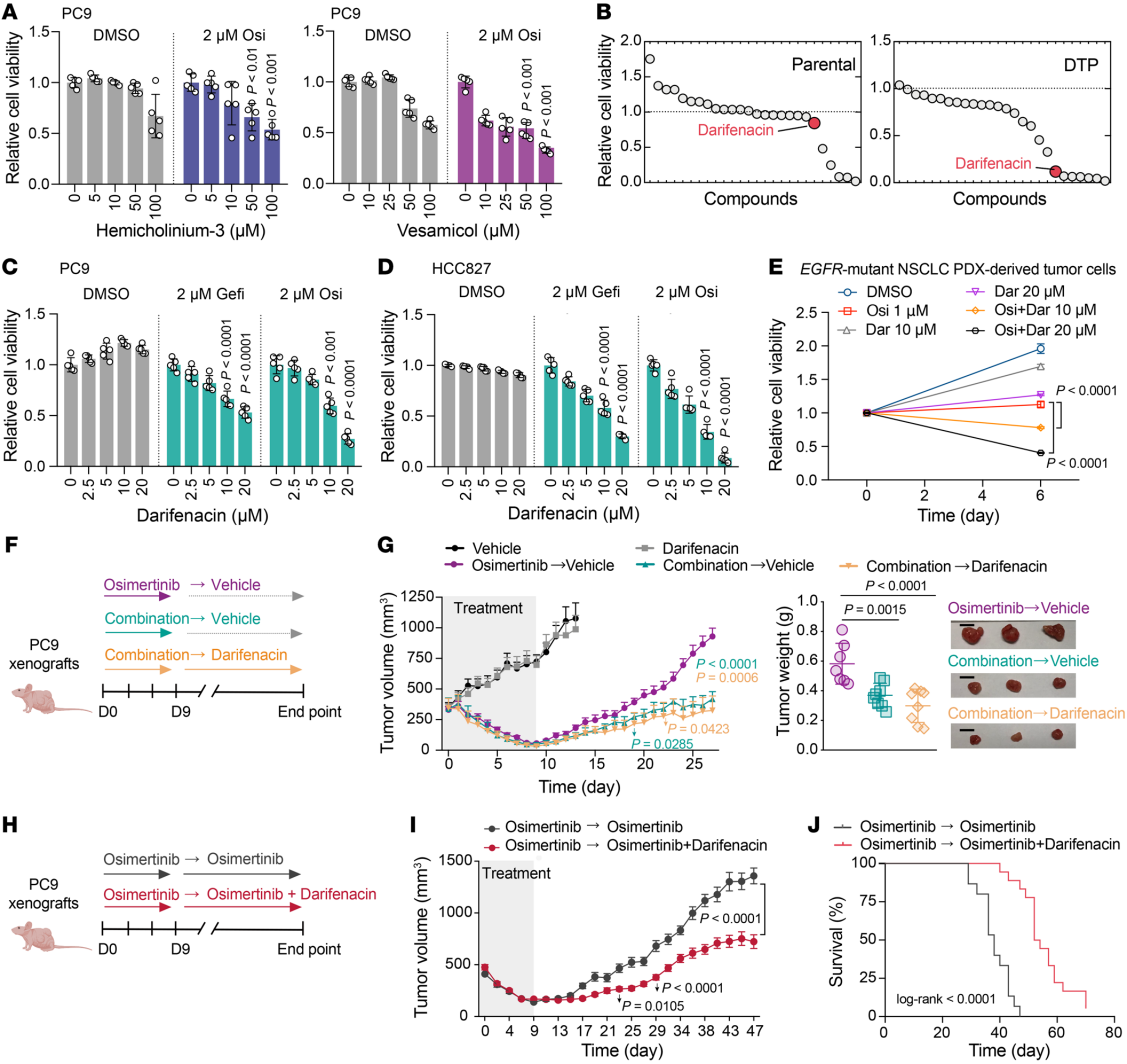
Pharmacological inhibition of ACh/M3R signaling suppresses DTP formation and retards tumor relapse. (**A**) Relative viability of PC9 cells cotreated with osimertinib and the indicated concentrations of hemicholinium-3 or vesamicol for 6 days (*n* = 5). (**B**) Drug screen in parental and PC9-derived DTP cells. Compounds were added in DMSO-containing (as parental cells group) or 2 μM EGFR-TKI–containing medium (as DTP cells group) for 6 days (*n* = 5). (**C** and **D**) Relative viability of cells cotreated with osimertinib or gefitinib and darifenacin for 6 days (*n* = 5). (**E**) Relative viability of tumor cells derived from the *EGFR*-mutant NSCLC645 PDX xenografts treated with osimertinib and darifenacin, alone or in combination for 6 days (*n* = 4 or 6). (**F** and **G**) PC9-xenograft mice were treated with 5 mg/kg osimertinib alone or in combination with 5 mg/kg darifenacin for 9 days, after which osimertinib was then withdrawn (osimertinib → vehicle, *n* = 7), combination of drugs was withdrawn (combination → vehicle, *n* = 8), or osimertinib was withdrawn and darifenacin treatment was continued (combination → darifenacin, *n* = 8) until the end of the experiment. Average tumor weights and image of relapsed tumors are shown on the right. Scale bars: 1 cm. (**H** and **I**) PC9-xenograft tumors were treated with 1 mg/kg osimertinib for 9 days followed by 1 mg/kg osimertinib (*n* = 15) or in combination with 5 mg/kg darifenacin (*n* = 18). (**J**) Percentage survival curve generated from PC9-xenograft mice that were treated with osimertinib alone (*n* = 15) or osimertinib for 9 days followed by combination with darifenacin (*n* = 18). In **A**, **C**–**E**, **G**, and **I**, data are shown as mean ± SEM. Significance was assessed using 1-way ANOVA with Dunnett’s test (**A**, **C**, and **D**), 2-way ANOVA with Dunnett’s test (**E**), 2-way ANOVA adjusted by Bonferroni’s correction (**G** and **I**), or 2-sided log-rank test (**J**).

Importantly, pharmacologically targeting ACh transport (vesamicol, a VAChT inhibitor) and targeting choline transport (hemicholinium-3, a choline transporter inhibitor) significantly reduced the extent of PC9-derived DTP formation (Figure 6A). Previous reports have demonstrated that ACh secreted by cancer cells can activate nAChR or mAChR signaling to promote cell proliferation (32, 45). To investigate whether blockade of ACh receptors decreases DTP formation, we screened small molecule inhibitors targeting nAChR and mAChR. Darifenacin is a potent inhibitor of M3R in clinical use and we found it significantly reduced the extent of PC9-derived DTP cell formation, doing so without killing parental PC9 cells (Figure 6, B and C, and Supplemental Figure 10, E and F). In addition, we observed that darifenacin reduced the extent of HCC827-derived DTP cell formation (Figure 6D). We next assessed the effects of darifenacin on drug tolerance in 3D culture models of tumor cells derived from *EGFR*-mutant PDX mice. We isolated single-cell suspensions from PDX tumor tissues to generate the 3D cultures, marking this isolation point as day 0, followed by treatment with osimertinib and/or darifenacin. Single-agent osimertinib treatment significantly inhibited cell survival on day 6 compared with the vehicle-treated group, but there were some osimertinib-tolerant cells formed. Moreover, cotreatment with osimertinib and darifenacin killed a higher proportion of the cultured cells, indicating reduced survival of osimertinib-tolerant cells (Figure 6E). Importantly, cotreatment with osimertinib and darifenacin heightened cell apoptosis compared with osimertinib treatment alone (Supplemental Figure 10G). Furthermore, interrupting ACh metabolism through *ChAT* knockdown with siRNA or pharmacological inhibition of M3R with darifenacin enhanced the sensitivity to osimertinib in all single-cell-derived PC9 clones (Supplemental Figure 11, A and B), demonstrating the important role of ACh metabolism and signaling in mediating single-cell-derived drug tolerance formation.

### ACh metabolism and signaling regulate the de novo and acquired resistance to EGFR-TKI.

Previous studies have demonstrated that the ACh pathway may mediate de novo resistance to EGFR-TKIs (46–49). To investigate the potential role of the ACh pathway in de novo (acute) resistance, we performed cell viability assays at early time points (48 and 72 hours) through genetic and pharmacological inhibition of ACh pathway modulators. We showed that treatment with vesamicol, hemicholinium-3, and darifenacin resulted in enhanced sensitivity to osimertinib inhibition (Supplemental Figure 12A). Overexpression of the short ChAT isoform led to decreased sensitivity to osimertinib at early time points (Supplemental Figure 12B). Conversely, knockdown of ACh pathway modulators ChAT, VAChT, and M3R conferred sensitivity osimertinib inhibition (Supplemental Figure 12C). To determine whether the ACh pathway regulates the acquired resistance to EGFR-TKIs, we used cells with acquired osimertinib resistance, which were established by culturing with increasing osimertinib concentrations for 6 months. Compared with parental cells, we found the short ChAT isoform was increased in osimertinib-resistant cells, which, however, was lower than that in DTP cells (Supplemental Figure 12D). An increased ACh level in osimertinib-resistant cells was also observed (Supplemental Figure 12E). In keeping with these observations, pharmacological inhibition of ACh pathways with hemicholinium-3, vesamicol, and darifenacin could resensitize the osimertinib-resistant PC9 and HCC827 cells to osimertinib (Supplemental Figure 12F). Consistently, knockdown of the ACh pathway regulators ChAT, VAChT, and M3R also resensitized osimertinib-resistant cells to osimertinib (Supplemental Figure 12G).

### Pharmacological blockade of ACh/M3R signaling delays tumor relapse in vivo.

After showing that disruption of ACh metabolism and its signaling pathway using pharmacological and genetic inhibition reduced DTP formation in vitro, we wanted to test in vivo whether the aforementioned FDA-approved M3R inhibitor darifenacin can prevent tumor relapse and prolong remission. Using PC9-xenograft mice, we tested the effects of darifenacin on MRD formation over a 9-day initial treatment phase, and also monitored darifenacin’s effects over a subsequent 18-day window representing the tumor relapse stage. Fourteen days after injection of the PC9 cells, mice were treated with darifenacin alone, osimertinib alone, or a combination of osimertinib and darifenacin (Figure 6F). Lacking an EGFR-TKI, it was unsurprising that treatment with darifenacin alone had minimal effects on tumor growth. As expected, treatment with osimertinib alone, as well as the combination of osimertinib and darifenacin, substantially regressed the tumor within 9 days (Figure 6G). To assess darifenacin’s specific impact on tumor relapse, the osimertinib treatment was withdrawn after day 9. As compared with the mice that received osimertinib alone, the mice treated with combination therapy showed delayed tumor relapse upon discontinuation of osimertinib, regardless of whether darifenacin therapy was continued thereafter or not (Figure 6G). Next, we assessed the levels of ACh and choline at the end point of in vivo experiments and only found a significant decrease in ACh in the combination-darifenacin group compared with the vehicle group, possibly because the long-term treatment with darifenacin both inhibited the emergence of DTP cells and eradicated the intrinsically high ChAT/ACh subpopulations (Supplemental Figure 13A). We observed only a slight decrease in choline levels in the regrown tumors in the osimertinib-vehicle group compared with the vehicle group, indicating that the regrown tumor, which relapsed quickly, may need to consume more choline for membrane lipid synthesis to support cell reproliferation (50) (Supplemental Figure 13A). Immunohistochemical analysis showed that the expression of ChAT, p-EGFR, and p-AKT was comparable among the different treatment groups; however, p-ERK was significantly inhibited in the darifenacin treatment groups (Supplemental Figure 13, B and C).

In additional experiments testing darifenacin’s effects on the formed MRD, PC9-xenograft mice received an initial treatment with osimertinib alone for 9 days to reduce the tumor size, thus allowing for the emergence of MRD. The mice were then randomly divided into 2 groups; one group was treated with osimertinib alone, whereas the other group was given a combined treatment of osimertinib with darifenacin (Figure 6H). As expected, the osimertinib-regressed residual tumors relapsed at a significantly reduced rate when treated with a combination of osimertinib and darifenacin compared with those treated with osimertinib alone (Figure 6I). Moreover, the mice given the combination therapy had significantly improved survival outcome compared with the mice given osimertinib alone (Figure 6J). Together, these in vivo results suggest that using combination therapies that include an EGFR-TKI alongside an agent to inhibit ACh/M3R signaling can decrease the establishment of residual tumors and retards tumor relapse.

### ACh mediates drug tolerance by inducing activation of WNT signaling.

**Figure 7:**
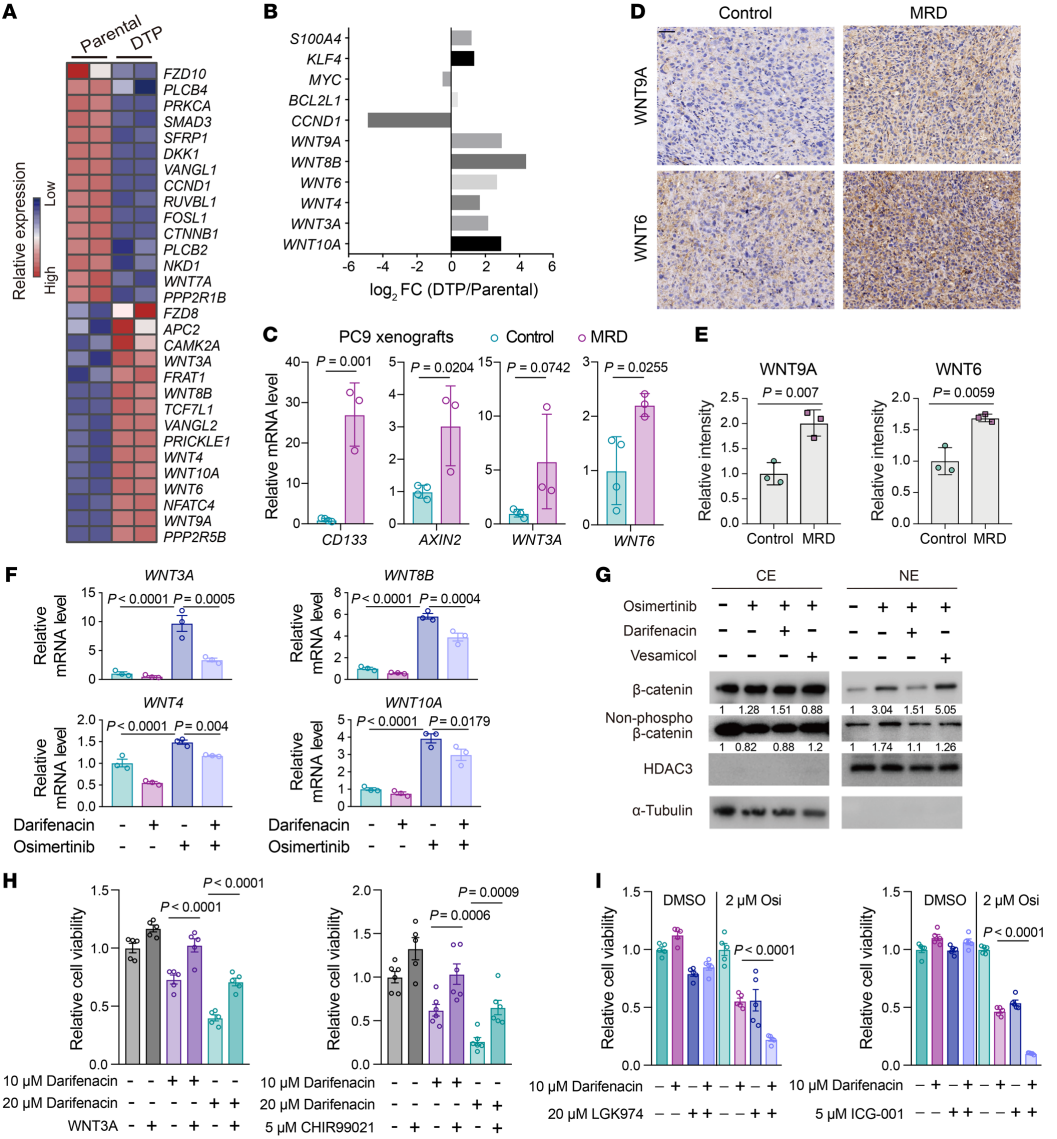
ACh modulates DTP cell formation through activating WNT signaling. (**A**) Heatmap of WNT signaling–related gene expression values in PC9-derived DTP versus parental PC9 cells (*n* = 2) with RNA-seq. Rows are *z* scores calculated for each gene in both cell types. (**B**) Log_2_-transformed fold change in the expression of WNT ligands and WNT target genes comparing PC9-derived DTP cells to parental PC9 cells with RNA-seq. (**C**) Relative mRNA levels of *CD133*, *AXIN2*, *WNT3A*, and *WNT6* in control (*n* = 4) and MRD (*n* = 3) PC9 xenografts. (**D** and **E**) Representative image and quantification of immunohistochemical staining of WNT6 and WNT9A on the indicated control and MRD tumor sections from PC9 xenografts. *n* = 3 mice per group. Scale bar: 50 μm. (**F**) Relative mRNA levels of WNT signaling–related genes in PC9 cells treated with osimertinib or indicated concentrations of darifenacin alone or in combination for 6 days (*n* = 3). (**G**) Western blot showing β-catenin and nonphosphorylated β-catenin in nuclear extract (NE) and cytosol extract (CE) of PC9 cells treated with 2 μM osimertinib or DMSO, alone or in combination with 20 μM darifenacin or 20 μM vesamicol. (**H**) Relative viability of PC9-derived DTP cells cotreated with darifenacin and WNT signaling activator CHIR99021 or WNT3A (*n* = 5 or 6). (**I**) Relative viability of PC9 cells treated with 2 μM osimertinib and indicated concentrations of darifenacin and WNT signaling inhibitor LGK974 or ICG-001, alone or in combination, for 6 days (*n* = 5). In **C**, **E**, **F**, **H**, and **I**, data are shown as mean ± SEM. Significance was assessed using 2-tailed Student’s *t* test (**C** and **E**) or 1-way ANOVA with Tukey’s test (**F**, **H**, and **I**).

To explore the mechanisms through which ACh drives drug tolerance, we revisited our RNA-seq data from parental PC9 cell and PC9-derived DTP cells. Notably, several WNT signaling molecules were among the upregulated genes in the DTP cells, including WNT ligands and WNT target genes. Consistent with activated WNT signaling, several known negative regulators of WNT signaling were among the downregulated genes in the DTP cells (Figure 7, A and B). WNT/β-catenin signaling has been linked to the formation of drug tolerance and residual tumor development (51, 52). In addition, qPCR analysis of osimertinib-regressed PC9 xenografts revealed that MRD tumors exhibited similar expression patterns for most of the WNT-related genes that were differentially expressed in DTP cells (Figure 7C). Furthermore, immunohistochemical analysis of osimertinib-regressed PC9 xenografts revealed elevated expression of WNT6 and WNT9A in MRD tumors compared with controls (Figure 7, D and E).

Given previous studies showing that ACh activates the muscarinic and WNT signaling pathways in intestines and gastric cancer (31), we hypothesized that ACh signaling may regulate the emergence of drug tolerance, potentially by activating WNT signaling. Indeed, cotreatment with osimertinib plus the M3R inhibitor darifenacin in parental PC9 cells significantly reduced the osimertinib-induced expression of WNT ligands and nuclear accumulation of β-catenin (Figure 7, F and G). In line with the observed pharmacological inhibition of ACh signaling, siRNA-mediated knockdown of ChAT significantly blocked the upregulated mRNA levels of WNT ligands in PC9 cells upon osimertinib treatment (Supplemental Figure 14A). In addition, overexpression of full-length ChAT upregulated WNT ligand expression (Supplemental Figure 14, B and C). By treating PC9 cells with ACh or ACh plus darifenacin, we observed that ACh heightened the nuclear accumulation of β-catenin, which could be blocked by darifenacin (Supplemental Figure 14D), and overexpression of full-length ChAT also resulted in the enhanced nuclear accumulation of β-catenin (Supplemental Figure 14E).

To further characterize the functional roles of ACh in mediating drug tolerance via regulation of WNT signaling, we incubated parental PC9 cells with osimertinib plus the aforementioned M3R inhibitor darifenacin, and then additionally treated them with various WNT signaling activators. Notably, the addition of WNT signaling activator CHIR99021 or WNT3A significantly reduced darifenacin’s capacity to reduce DTP formation (Figure 7H). We therefore speculated that blocking WNT signaling with a small molecule inhibitor might further enhance darifenacin’s capacity to reduce DTP formation. Indeed, the administration of darifenacin alongside WNT signaling inhibitors LGK974 and ICG-001 caused a further inhibition of DTP formation compared with darifenacin treatment alone (Figure 7I). Collectively, these findings confirm that the EGFR-TKI–induced ACh increase mediates the emergence of drug tolerance by activating WNT signaling.

Previous studies have demonstrated the overlapping biological processes and cross-regulation between YAP/TAZ and WNT/β-catenin signaling (53–55). Notably, YAP/TAZ plays both positive and negative roles in the WNT signaling pathway, which is highly correlated with the cellular localization of YAP/YAZ (56–58). Here, we found that YAP silencing or treatment with the YAP/TEAD inhibitor verteporfin could attenuate the increase in several WNT ligands induced by osimertinib treatment, including WNT3A, WNT4, and WNT8B, which was partially rescued by exogenous ACh supplementation (Supplemental Figure 15, A and B), suggesting that YAP could regulate the expression of several WNT family members under osimertinib treatment.

### Perturbed ACh metabolism correlates with drug response to EGFR-TKI in human NSCLC patients.

**Figure 8:**
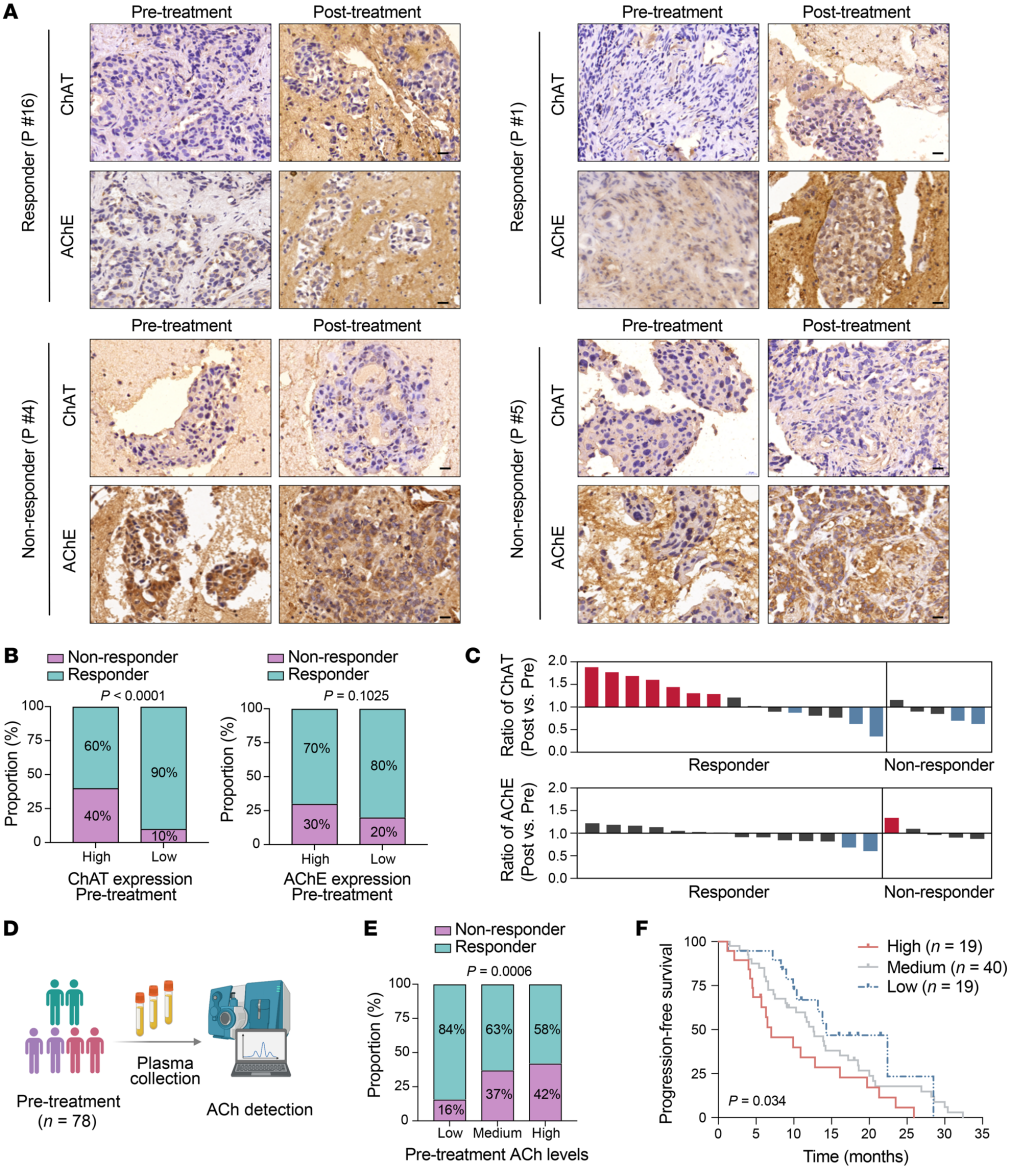
Tumor ChAT expression and plasma ACh levels correlate with drug response to EGFR-TKI in human NSCLC patients. (**A**) Representative image of immunohistochemical staining of ChAT and AChE on tumor sections collected from human NSCLC patients before and after EGFR-TKI treatment. Scale bar: 20 μm. (**B**) The proportion of responders and nonresponders to EGFR-TKI among patients according to ChAT and AChE levels (high, top 50%; low, bottom 50%) before EGFR-TKI treatment. (**C**) Changes in ChAT and AChE on tumor sections collected from human NSCLC patients before and after EGFR-TKI treatment. Ratios of ChAT and AChE levels (Post versus Pre) with significant changes were labeled as red (increase) and blue (decrease), or others labeled as black. (**D**) Pretreatment plasma samples were collected from human NSCLC patients for ACh detection. The illustration was created with BioRender.com. (**E**) The proportion of responders and nonresponders to EGFR-TKI among patients with low (bottom 25%, *n* = 19), medium (medium 50%, *n* = 40), and high (top 25%, *n* = 19) levels of pretreatment plasma ACh. (**F**) Kaplan-Meier survival of patients with high, medium, and low levels of pretreatment plasma ACh. Significance was assessed using the χ^2^ test (**B** and **E**) or Gehan-Breslow-Wilcoxon test (**F**).

To determine the clinical relevance of the key regulators ChAT and AChE in ACh metabolism, we performed immunohistochemical analysis on 20 paired tumor biopsies taken before and after EGFR-TKI therapy. Interestingly, we found that before treatment, high ChAT levels significantly correlated with worse response. However, no significant correlation between pretreatment AChE levels and clinical therapy response was observed (Figure 8, A and B). DTP/MRD cells are found to be mostly enriched in patients who achieve complete response and/or partial response (14, 16). A previous study has revealed that an increase in the neural crest stem–like cell gene expression signature, a marker for the DTP cell population, could be only detected in some of the patients (20%) with partial response to targeted therapy in BRAF-mutant melanoma but was absent from patients with progressive disease and/or stable disease (17). Consistently, our result showed that all the patients who exhibited an increase in ChAT after EGFR-TKI were responders (partial response). However, the nonresponders with de novo resistance (progressive disease and/or stable disease, without residual disease formation) did not exhibit the increased ChAT expression upon EGFR-TKI, possibly because DTP cells may not emerge and be enriched in nonresponders during treatment (Figure 8C). Thus, these data suggest that the regulation of ChAT after EGFR-TKI was highly correlated with initial clinical response of patients. A ChAT increase may serve as an indicator that the TKI is initially effective in inhibiting EGFR, which may also indicate residual disease formation and the origin of tumor recurrence at a late stage. Notably, 85% of patients (17 out of 20) showed no apparent change in AChE after treatment, in line with our Western blotting and immunohistochemical data from cell lines and mouse models showing a negligible change in AChE levels upon EGFR-TKI treatment (Figure 8C). Hence, these data suggest that the accumulation of ACh in the DTP state may primarily be attributed to the upregulation of ChAT.

ACh, as a neurotransmitter, is mainly secreted by the autonomic nervous system but also by some cancer cells such as squamous cell lung carcinoma and hepatocellular carcinoma (45, 59). We conducted additional in vitro experiments with PC9 cells and observed, as expected, significantly increased secretion of ACh from PC9-derived DTP cells into the culture medium compared with parental cells (Supplemental Figure 15C). Next, we wondered whether plasma ACh levels were associated with patients’ clinical response to EGFR-TKIs. We performed a cohort study to examine the pretreatment plasma ACh levels of NSCLC patients (*n* = 78) with *EGFR*-mutant tumors (Figure 8D). In order to assess the correlation between pretreatment plasma ACh levels and clinical EGFR-TKI efficacy, we divided the patients into low (*n* = 19, bottom 25%), medium (*n* = 40, middle 50%), and high (*n* = 19, top 25%) groups based on baseline ACh levels in pretreatment samples. Interestingly, the response rate for EGFR-TKIs in patients with low ACh levels was relatively high (84%), whereas for those patients with high and medium ACh levels, the response rate was relatively low (58% and 63%, respectively) (Figure 8E), demonstrating the pretreatment plasma ACh levels in patients may inversely correlate with clinical response rate. Furthermore, we also found that patients with high pretreatment ACh levels showed a shorter progression-free survival compared with patients with low and/or medium pretreatment ACh levels (Figure 8F). Collectively, high ChAT expression in tumors and/or high plasma ACh levels before EGFR-TKI highly correlated with worse response, which was in line with the in vitro observations that exogenous ACh and ChAT overexpression reduced the drug sensitivity to EGFR-TKI of parental cells, suggesting the correlation between activated ACh metabolism and signaling and de novo resistance.

## Discussion

Increasing research attention has focused on understanding the impacts of DTP cell subpopulations — which survive initial therapies via nonmutational adaptions — on the long-standing problem of cancer treatment resistance (4, 8, 9, 22). Our study suggests that metabolic reprogramming for the neurotransmitter ACh is one such nonmutational adaptive mechanism that underlies the emergence of therapy-induced drug tolerance and residual disease. We also show that EGFR-TKI–dependent DTP cell formation is transient and reversible. Altering ACh biosynthesis and M3R signaling regulates DTP formation in part through suppression of WNT signaling. Excitingly, the combination of EGFR-TKI with targeting ACh/M3R signaling is shown to retard tumor relapse and achieve a long-lasting drug response in vivo. Additionally, plasma ACh levels and tumor ChAT expression highly correlated with response to EGFR-TKI and progression-free survival in human NSCLC patients. Notably, several studies have shown that altered metabolic pathways in cancers have marked effects on therapeutic outcomes (10, 18–21, 60), and our findings define a metabolic vulnerability in drug tolerance that can be therapeutically exploited to prevent cancer drug resistance and tumor relapse.

It is well established that ACh, the first identified prototypical neurotransmitter, plays numerous nonneuronal roles, including modulating the cytoskeleton, induction of vasodilation, antiviral immunity, and tumorigenesis (31, 61–63). In particular, several kinds of cancer cells, such as small cell lung carcinomas (32), hepatocellular carcinomas (45), and gastric carcinomas (64) have been shown to synthesize and secrete ACh and then respond to endogenous ACh for proliferation, thus creating a functional cholinergic autocrine loop. Although many cancers utilize ACh as an autocrine or paracrine growth factor, its role in modulating drug resistance is less well understood. We reveal that EGFR-TKI–induced DTP cells are highly dependent on the upregulated ACh biosynthetic process, and interference with ACh metabolism and its signaling pathway can dramatically inhibit DTP formation. Indeed, these intriguing findings shed light on the previously unrecognized role of activated autocrine ACh signaling in driving initial cancer therapy escape, which exposes a therapeutic vulnerability that can be clinically exploited to prolong the efficacy of EGFR-TKI treatment. Moreover, the correlation between plasma ACh levels and EGFR-TKI response in human NSCLC patients suggests the ACh level in peripheral blood may predict clinical outcomes and help stratify patients to define an appropriate targeting strategy, although the origin of ACh in blood is diverse and remains to be elucidated. Notably, extensive studies have implicated the importance of the central nervous system and tumor-associated nerve networks in modulating cancer progression and metastasis through connecting neurons to cancer cells via neurotransmitters (26–29). For example, perineural invasion–derived ACh accumulation could promote tumor growth through eliciting an immunosuppressive microenvironment in pancreatic ductal adenocarcinoma (65). Since solid tumors are highly innervated by cholinergic fibers, it is necessary to further investigate crosstalk between nerve fibers in the tumor environment and tumor cells upon anticancer therapy and the potential modulatory role of nerve fibers in the initial therapy evasion. Interestingly, a very recent study has demonstrated that the neurotransmitter GABA could be synthesized and secreted by B cells and modulate the antitumor immune response (33). In fact, many immune cells, including T cells, B cells, and macrophages, have also been reported to synthesize and release ACh, which exerts immunoregulatory functions (63, 66–68). It is noteworthy that hijacking the microenvironment is the main nonmutually exclusive strategy deployed by DTP cells to survive (69, 70). The nontumor cells existing in the microenvironment, including cancer-associated fibroblasts (71), neutrophils (72), and tumor-associated macrophages (73) could secrete cytokines, chemokines, and metabolites to support DTP cell survival upon anticancer treatment. In the future, it would be worth exploring whether the tumor cell–derived ACh could affect the immune microenvironment.

Aberrant activation of the WNT/β-catenin pathway is associated with drug resistance and acquisition of neuroendocrine features in prostate cancer (74, 75). Additionally, in NSCLC and basal cell carcinomas, WNT signaling has been reported to modulate drug tolerance. For example, pharmacological blockade of WNT signaling with LGK974, which inhibits WNT ligand secretion through the acyltransferase porcupine, or with an antibody against the WNT receptor LRP6 leads to a reduction in the number of DTP cells and a delay of tumor relapse upon drug withdrawal (51, 52, 76). Although LGK974 has already been assessed in clinical trials (77), the side effects associated with the complete blockade of the WNT pathway represent a huge challenge in the development of an effective and safe therapeutic strategy because of the crucial role of this pathway in physiological homeostasis (78). Nevertheless, our mechanistic studies delineate that EGFR-TKI–induced ACh release from DTP cells drives downstream induction of WNT ligands and nuclear translocation of β-catenin through stimulation of muscarinic signaling, thus activating the WNT pathway and promoting DTP cell survival. Functionally, WNT signaling activators partially alleviate the suppression of DTP formation caused by ACh signaling inhibition. These observations suggest that targeting the ACh signaling pathway, which is pharmacologically manipulatable, may represent a more druggable strategy for targeting the WNT pathway. Strikingly, our data indicated that the disruption of ACh signaling using the M3R antagonist darifenacin, which is clinically approved for treating overactive bladder (79), suppresses the emergence of residual DTP cells in vitro and in vivo. It may be feasible to rapidly advance this combination therapy into clinical trials.

We acknowledge that some limitations exist in this study. First, we demonstrated the accumulation of ACh in osimertinib-regressed PDX tumors, but we were not able to validate these phenotypes in clinically fresh specimens because osimertinib-regressed tumors from NSCLC patients upon EGFR-TKI therapy are not readily accessible. In addition, the reduction in PDX tumor size upon osimertinib treatment was modest, which possibly resulted from the discrepancy in the drug response correlated to the distinct clinical behaviors of patients. It is extremely necessary to establish more PDX models and monitor the longitudinal drug response, and further explore the metabolic trajectory from drug-naive, MRD, and regrown tumors in future work. Second, in the in vivo experiments, we used darifenacin, an M3R inhibitor that has in vivo stability and a high degree of M3R selectivity (79). Notably, it has been reported that similar plasma darifenacin concentrations can be achieved in nude mice receiving 3 mg/kg/day and in human patients taking 15 mg to 30 mg darifenacin once per day (80). Hence, we treated mice with a clinically relevant dose, 5 mg/kg/day of darifenacin, and found no significant change in mouse body weight. In addition, no locomotion/movement or additional signs of autonomic dysregulation were observed, indicating a relatively safe dosage used in our study with limited negative side effects. However, our data do not allow us to exclude potential nonspecific effects induced by drug-drug interactions when darifenacin is dosed in combination with EGFR-TKIs. Thus, further investigation is necessary to examine the efficacy and safety of the combination therapy of EGFR-TKIs with darifenacin in clinical trials.

In summary, we identify an EGFR-TKI–induced ACh metabolism reprogramming pathway in DTP cells, which display WNT signaling activation, an important contributor to drug tolerance. Moreover, this metabolic adaptation during initial EGFR-TKI therapy implies that targeting the ACh/M3R/WNT axis may represent a promising therapeutic modality to improve the efficacy of existing therapies. Taken together, our study provides a perspective for the design of more effective and personalized intervention strategies for patients receiving EGFR-TKI therapy.

## Methods

A detailed description of the materials and methods used are provided in the Supplemental Materials and Methods.

### Statistics.

All statistical methods used are listed in the figure legends or corresponding methods. In brief, all grouped data are presented as mean ± SEM unless stated otherwise. Statistical analyses were performed with GraphPad Prism v.7.0 and R v.3.6.0 software (https://www.r-project.org/). IC__50__ values were determined using a nonlinear regression analysis fit of the normalized dose-response curves. A 2-tailed Student’s *t* test was used to compare 2 groups of independent samples. One-way or 2-way ANOVA with Dunnett’s test or Tukey’s test was used for analyses of more than 2 groups. The in vivo data were analyzed by 2-way ANOVA with Bonferroni’s correction to adjust the significance level for multiple comparisons. Kaplan-Meier survival curves for TCGA lung adenocarcinoma and lung squamous cell carcinoma patients with EGFR somatic mutations with different ChAT levels and clinical patients with different plasma ACh levels were compared by 2-sided log-rank test. A *P* value or adjusted *P* value of less than 0.05 was considered statistically significant.

### Study approval.

All animal protocols related to mouse experiments were approved by the University of Tsinghua Institutional Animal Care and Use Committee. The animal protocols are compliant with all relevant ethical regulations. All human studies were conducted according to the principles of the Declaration of Helsinki, and approved by the Institutional Review Board and Biomedical Ethics Committee of Shanghai pulmonary hospital of Tongji University, Affiliated Tumor Hospital of Guangxi Medical University, and West China Hospital of Sichuan University. Written informed consent was obtained from all patients.

## Author contributions

MN and ZH conceived the project, designed the study, and wrote the paper. RJD assisted in editing the manuscript. MN designed, performed, and analyzed the in vitro and in vivo experiments and interpreted the results of NSCLC cell line xenograft model and PDX model. NC performed and analyzed the experiments related to NSCLC cell lines and xenografts. HP performed the metabolomics experiments. LY and YC assisted with cell culture experiments. TJ, WJ, QY, PT, WL, and CZ provided the clinical samples. ZH supervised the project.

## Supplementary Material

Supplemental data

## Version 1 09/01/2022

In-Press Preview

## Version 2 10/17/2022

Electronic publication
