# Supplementary material for: Targeting acetylcholine signaling modulates persistent drug tolerance in EGFR-mutant lung cancer and impedes tumor relapse
Source: J Clin Invest. 2022 Oct 17;132(20):e160152. doi: 10.1172/JCI160152 (PMC9566900; doi:10.1172/JCI160152)
Supplement: Supplemental data [file jci-132-160152-s134.pdf]

Supplemental Information

Targeting acetylcholine signaling modulates persistent drug tolerance in EGFR-mutant lung cancer and impedes tumor relapse

Meng Nie, Na Chen, Huanhuan Pang, Tao Jiang, Wei Jiang, Panwen Tian, LiAng Yao, Yangzi Chen, Ralph J. DeBerardinis, Weimin Li, Qitao Yu, Caicun Zhou, and Zeping Hu

Supplemental Materials and Methods

Supplemental Figures 1-15

Supplemental Tables 1-4

Materials and methods

Cell culture and generation of drug-tolerant persister and acquired resistance cells. Human NSCLC cell lines PC9, HCC827, H1975 were obtained from ATCC and lung fibroblast cell line MRC-5 was obtained from Cell Bank of Chinese Academy of Sciences. PC9, HCC827 and H1975 were maintained in RPMI-1640 medium and MRC-5 was maintained in MEM medium, which were supplemented with penicillin/streptomycin, and 10% fetal bovine serum (FBS) at 37°C in a humidified atmosphere containing 5% CO_2 and 95% air. All cells were routinely screened for the absence of mycoplasma contamination and were validated by short tandem repeat (STR) DNA fingerprinting. DTP cells were generated through continuous treatment with 2 μM of Gefitinib, Erlotinib, Osimertinib or Rociletinib for 9 days, according to protocols described previously (1). Acquired resistant cells were generated by treating parental cells with increasing concentrations of Osimertinib starting at 10 nM, which was followed by a stepwise dose increase every 72 h up to 1 μM.

Chemicals. Osimertinib (S7297), Gefitinib (S1025), Erlotinib (S7786), Rociletinib (S7284), LGK974 (S7143), CHIR99021 (S2924) and ICG-001 (S2662) were purchased from Selleck Chemicals. D-(+)-Vesamicol hydrochloride (V104), Hemicholinium-3 (H108) and Acetylcholine chloride (A2661) were purchased from Sigma-Aldrich. Verteporfin (HY-B0146) was purchased from MedChemExpress. Compounds used for drug screen were listed below: Darifenacin HBr (S3144), Ipratropium Bromide (S1683), Oxybutynin (S1754), Irsogladine (S1929), Methscopolamine (S1978), Atropine sulfate monohydrate (S2130), Scopolamine HBr (S2508), Pancuronium dibromide (S2497) were purchased from Selleck Chemicals. Gallamine triethiodide (G8134), DL-Homatropine hydrobromide (H0126), *p*-Fluorohexahydro-sila-difenidol hydrochloride (H127), Hexamethonium bromide (H0879), MG624 (M3184),

Mecamylamine hydrochloride (M9020), Methoctramine hydrate (M105), Orphenadrine hydrochloride (O3752), Pirenzepine dihydrochloride (P7412), Propantheline bromide (P8891), Succinylcholine chloride dihydrate (S8251), DL-Trihexyphenidyl hydrochloride (T1516), Tetraethylammonium chloride (T2265), Telenzepine dihydrochloride hydrate (T122), Tropicamide (T9778), Benztropine mesylate (B8262), Biperiden hydrochloride (B5311), and 4-DAMP methiodide (D104) were purchased from Sigma-Aldrich.

Analysis of sequence variations. DNA was extracted from parental cells and Osimertinib-tolerant cells from the PC9 cell line (TaKaRa) and sequenced by the Sanger method. DNA fragments containing EGFR exons 19 and exons 20 were amplified with primers EGFR-19 forward, 5’-CAATATCAGCCTTAGGTGCGGC-3’; EGFR-19 reverse, 5’-AACATTTAGGATGTGGAGATGAGC-3’; EGFR-20 forward, 5′-ATGGCCAGCGTGGACAAC-3′; EGFR-20 reverse 5′-ATCTCCCTTCCCTGATTAC-3′. PCR reaction was performed at 94°C for 5 min; 35 cycles of 94°C for 30 s, 56°C for 30 s and 72°C for 40 s; and 72°C for 7 min. The PCR products were bidirectional sequenced on ABI 3730 XL sequencers (Applied Biosystems) with ABI BigDye Terminator v3.1 Cycle Sequencing Kits and analyzed by Chromas Sequence Scanner Software.

Cell cycle analysis. PC9 cells were seeded at 3×10^5 cells / well in 6-well plastic culture plates at day 0. Then, 2 μM Osimertinib was added on the following day. Plates were incubated at 37°C in 5% CO_2 for 96 h. Following treatment, cells were stained with Propidium Iodide (Beyotime) following manufacturer instructions and analyzed by flow cytometry (BD Calibur).

RNA sequencing. Total RNA from approximately 1 million Osimertinib-tolerant PC9 cells or parental PC9 cells was isolated by Trizol (Life Technologies) following the manufacturer’s

instructions. cDNA libraries were sequenced on an Illumina HiSeqX-ten to obtain 150-bp pair-end sequence reads. Sequencing quality was evaluated by FastQC v.0.11.8. FASTQ files were aligned to hg38 (GRCh38) using STAR v.2.6.1b (default parameters). Gene level counts were computed using featureCounts v.1.6.3 with the same human genome reference used for mapping (Gencode v.27). Differential expression analysis was performed with DESeq2 v.1.22.2 in R, and a threshold of fold change > 2 and adjusted p value < 0.05 was used to call differentially expressed genes. KEGG pathway enrichment was analyzed using DAVID v.6.7 (https://david-d.ncifcrf.gov). Gene Ontology term enrichment was performed using Metascape (http://metascape.org). Only terms with $p < 0.01$ and with at least 3 enriched genes were considered as significant. Heatmaps were generated using pheatmap v.1.0.12 in R. For GSEA enrichment analysis, the Molecular Signatures Database (MSigDB) was used as a source of gene sets. The enrichment of signatures was assessed using the Gene Set Enrichment Analysis (GSEA) v.4.0.2. Genes were ranked by Diff_of_classes parameter. Gene sets used were listed in Supplemental Table 1. Enrichment scores were corrected for gene-set size (normalized enrichment score). The p value estimates the statistical significance of the enrichment score for a single gene set. The RNAseq data in our study have been deposited into the NCBI Gene Expression Omnibus (GEO) database under accession number GSE153183.

Metabolomics. PC9 and HCC827 derived-DTP cells were induced as protocols described above. Two hours before collection, cells were incubated with fresh medium. At the time of collection, cells were washed with ice-cold saline, lysed with 80% methanol in water and quickly scraped into Eppendorf tubes. The insoluble material was pelleted in a cooled centrifuge at 4°C and the supernatant was transferred to a new tube and evaporated to dryness using a SpeedVac concentrator (Thermo Scientific). Metabolites were reconstituted in LC-MS grade water with

0.03% formic acid, vortex-mixed and centrifuged to remove debris. For PC9 cell xenografts and NSCLC patient-derived xenografts metabolomics experiments, tumor samples were homogenized in 80% methanol in water and centrifuged at 15,000 rpm for 15 min at 4°C. The supernatant was transferred to a new tube and evaporated to dryness as described above for cell lysates. Samples were randomized and blinded before analyzing by LC-MS/MS. Chromatographic separation was performed on a Nexera Ultra High-Performance Liquid Chromatograph (UHPLC) system (Shimadzu), with a RP-UPLC column (HSS T3, 2.1 mm × 150 mm, 1.8 µm, Waters) and the following gradient: 0-3 min 99% mobile phase A; 3-15 min 99-1% A; 15-17 min 1% A; 17-17.1 min 1-99% A; 17.1-20 min 99% A. Mobile Phase A was 0.03% formic acid in water. Mobile Phase B was 0.03% formic acid in acetonitrile. The flow rate was 0.25 $mL \cdot min^{-1}$, the column was at 35°C and the samples in the autosampler were at 4°C. Mass spectrometry was performed using an AB QTRAP 6500+ triple quadrupole mass spectrometer (Applied Biosystems SCIEX) in multiple reaction monitoring (MRM) mode for the detection of 256 unique endogenous water-soluble metabolites as we previously described, with some modifications (2, 3). Chromatogram review and peak area integration were performed using MultiQuant 3.0.2 (Applied Biosystems SCIEX), and the peak area of each detected metabolite was normalized to the sum of peak areas of all detected metabolites in that sample to correct for any variations introduced by sample handling through instrument analysis. The normalized data were used as variables for the multivariate and univariate statistical analysis. Metabolomics data analysis and hierarchical clustering were performed using Metaboanalyst v.3.0 (http://www.metaboanalyst.ca) and Simca v.14.1 (Umetrics). Univariate statistical differences of the metabolites between two groups were analyzed using a two-tailed Student's *t*-test. t-SNE scatterplots were generated of metabolomics data of PC9 xenografts using R Rtsne v0.15

package. Clustering for the control, MRD, and regrown tumors was performed using detected metabolites by R Mfuzz v2.44.0 package.

Quantitative detection of ACh by mass spectrometry. To measure ACh and choline in cells, cell culture medium, tumor tissues from xenografts and NSCLC patient-derived PDX as well as human plasma, Trimethyl-D9-choline was added to the 80% methanol in water as an internal standard (IS) to achieve the final concentration of 5 nM. A normal-phase UPLC (NP-UPLC) method was developed using the above-mentioned UHPLC system on a NP-UPLC column (BEH HILIC, 2.1 mm × 100 mm, 1.7 μm, Waters) with the following gradient: 0-3 min 99% mobile phase A; 3-15 min 99-1% A; 15-17 min 1% A; 17-17.1 min 1-99% A; 17.1-20 min 99% A. Mobile Phase A was 5 mM NH_4COOH in H_2O: ACN (5: 95). Mobile Phase B was 5 mM NH_4COOH in H_2O: ACN (50: 50). The flow rate was 0.5 $mL \cdot min^{-1}$, the column was at 35°C and the samples in the autosampler were at 4°C. Mass spectrometry was performed using an AB QTRAP 6500+ triple quadrupole mass spectrometer (Applied Biosystems SCIEX) in MRM positive mode for the detection of ACh (146.0/87.0), choline (104.0/60.0), and IS (113.3/69.2). Chromatogram review and peak area integration were performed using MultiQuant software version 3.0.2. The concentrations of analytes were calculated by peak area ratios of the analytes to IS using standard curves generated with weighted linear regression analysis in MultiQuant 3.0.2. Statistical differences of the metabolites between the two groups were analyzed using a two-tailed Student's *t*-test.

Patient samples. All human studies were conducted according to the principles of the Declaration of Helsinki, and approved by the Institutional Review Board and Biomedical Ethics Committee of Shanghai pulmonary hospital of Tongji University, Affiliated Tumor Hospital of Guangxi Medical University, and West China Hospital of Sichuan University. Pre-EGFR TKI

treatment plasma samples were obtained from Shanghai pulmonary hospital of Tongji University, Affiliated Tumor Hospital of Guangxi Medical University, and West China Hospital of Sichuan University. Tumor and normal adjacent tissues from NSCLC patients with or without *EGFR* mutation were obtained from Shanghai pulmonary hospital of Tongji University. Detailed patient clinical data are listed in Supplemental Table 2 and Supplemental Table 3.

Cell viability and apoptosis assays. Briefly, tumor cells (2-5 × 10^3 cells / 100 μL / well) in RPMI 1640 medium supplemented with 3% or 10% FBS were plated in 96-well or 384-well plates and cultured with drugs. Cell viability was assessed using CellTiter Glo Luminescent Cell Viability assay (Promega). For DTP formation assays, the cells were treated with or without EGFR TKIs in combination with indicated compounds for 6 days. The remaining cell viability was measured for each parental and DTP cells model. For cell apoptosis assay, 1×10^5 cells were stained with FITC Annexin V and PI from an apoptosis detection kit (Beyotime, C1062) and analyzed for apoptosis by flow cytometry on an LSRFortessa SORP (BD) according to the manufacturer's recommendations.

Drug screen. We performed a drug screen using 26 small molecule compounds (detailed information of chemicals was list in the Supplemental Table 4) that targeting mAChR and nAChR. In brief, compounds were added in DMSO-containing (as parental cell group) or 2 μM EGFR TKI-containing medium (as DTP cell group). After 6 days of incubation, each well was measured using CellTiter Glo reagent (Promega). All compounds from the screen library were tested at six concentrations. The screens were performed in 6 replicates. The relative survival of parental PC9 cells and EGFR TKI-induced tolerant PC9 cells in the presence of compounds was normalized against negative control conditions (compounds-untreated cells).

Colony-formation assays. Cells were cultured and seeded into 6-well plates at a density of 3-5 × 10^4 cells / well and were cultured in medium containing the indicated drugs for 9 days. The medium was replenished every 3 days. Cells were fixed with 4% formaldehyde in PBS and stained with 0.5% crystal violet (Leagene). To evaluate the effect of exogenous ACh on DTPs formation, PC9 and HCC827 cells were pretreated with exogenous ACh for 3 and/or 9 days, followed by the combination with the 2 μM Osimertinib for 9 days. Images were analyzed using ImageJ with ColonyArea plugin.

RT-PCR and real-time PCR. Total RNA from cells and tumor samples was isolated using Trizol (Life Technologies) according to the manufacturer's instructions. cDNA synthesis was performed using PrimeScript™ RT reagent Kit from TaKaRa. RT-PCR reactions were performed with 2x Taq Master Mix (Vazyme). Quantitative real-time PCR reactions were performed with iTaq Universal SYBR green Supermix with blended ROX and fluorescein passive reference dyes from Bio-Rad. The experiments were performed according to the manufacturer's instructions. The sequences of the primers used for RT-PCR analyses were as follows:

common ChAT forward, 5'-GGAGATGTTCTGCTGCTATG-3'; common ChAT reverse, 5'-GGAGGTGAAACCTAGTGGCA-3';

ChAT-212 transcript forward, 5'-GTTTCCCCCATGCTGTTCT-3'; ChAT-212 transcript reverse, 5'-TACAAGGCAGATGCAGCGC-3'.

GAPDH forward, 5'-GGAGCGAGATCCCTCCAAAAT-3'; GAPDH reverse, 5'-GGCTGTTGTCATACTTCTCATGG-3'.

The sequences of the primers used for quantitative real-time RT-PCR analyses were as follows:

common ChAT forward, 5'- CATCGTCCTGGTGCAGTG -3'; common ChAT reverse, 5'-GAAGTTTTTCTGCCGAGGAG -3'; ChAT-212 transcript forward, 5'-GTTTCCCCCATGCTGTTCT -3'; ChAT-212 transcript reverse, 5'-ATCGAATGGGGAGTGTTCG-3'; CD133 forward, 5'-TTACGGCACTCTTCACCT-3'; CD133 reverse, 5'-TATTCCACAAGCAGCAAA-3'; AXIN2 forward, 5'-TACACTCCTTATTGGGCGATCA-3'; AXIN2 reverse, 5'-TTGGCTACTCGTAAAGTTTTGGT-3'; WNT3A forward, 5'-TCAGCTGCCAGGAGTGCACG-3';WNT3A reverse, 5'-CGCCCTCAGGGAGCAGCCTAC-3'; WNT10A forward, 5'-CCCAATGACATTCTGGACCT-3'; WNT10A reverse, 5'-TAAGCGGTGCAGCTTCCTAC-3'; WNT5A forward, 5'-ATGAACCTGCACAACAACGA-3'; WNT5A reverse, 5'-CTTCTCCTTCAGGGCATCAC-3'; WNT4 forward, 5'-GCAGAGCCCTCATGAACCT-3'; WNT4 reverse, 5'-CACCCGCATGTGTGTCAG-3'; WNT8B forward, 5'-CCGACACCTTTCGCTCCATC-3'; WNT8B reverse, 5'-CAGCCCTAGCGTTTTGTTCTC-3'; WNT9A forward, 5'- AGCAGCAAGTTCGTCAAGGAA -3'; WNT9A reverse, 5'- CCTTCACACCCACGAGGTTG -3'; BCL2L1 forward, 5'-GAGCTGGTGGTTGACTTTCTC-3'; BCL2L1 reverse, 5'-TCCATCTCCGATTCAGTCCCT-3'; S100A4 forward, 5'-AACTAAAGGAGCTGCTGACCC-3'; S100A4 reverse, 5'-TGTTGCTGTCCAAGTTGCTC-3'; WNT6 forward, 5'-AGAGTGCCAGTTCCAGTTCC-3'; WNT6 reverse, 5'-GAACACGAAGGCCGTCTC-3'; GAPDH forward, 5'-ACCCAGAAGACTGTGGATGG-3'; GAPDH reverse, 5'-TTCAGCTCAGGGATGACCTT-3'.

RNA interference. For siRNA transfection, lipofectamine RNAiMAX (Thermo) was used according to the standard protocol. All siRNAs targeting the genes were obtained from Genepharm Technologies. The two ChAT siRNA targeting sequences were 5'-

GGUAUGGUGCCUGCUACAATT-3’ and 5’-AGAUGUUCAUGGAUGAAACTT-3’. pLV vectors expressing shRNAs targeting the mRNA sequences of human ChAT (shRNA1: pHS-ASR-0428; shRNA2: pHS-ASR-0429) and corresponding empty vector (shRNA: pHS-ASR-LW429) were purchased from Syngentech; pLKO vectors expressing shRNAs targeting M3R (shRNA1: TRCN0000011259, shRNA2: TRCN0000011260), VAChT (shRNA1: TRCN0000044606, shRNA2: TRCN0000044607), YAP (shRNA1: TRCN0000107265, shRNA2: TRCN0000107268) and control shRNA (SHC002) were purchased from the Centre of Biomedical Analysis, Tsinghua University, shRNA library. psPAX2 and pMD2.G were purchased from Addgene (Cambridge), Opti-MEM was purchased from Gibco, and Lipofectamine 3000 was purchased from Invitrogen. The ratios used were recombinant lentivirus plasmid: psPAX2: pMD2.G = 2:1:1 and plasmid: Lipofectamine = 1:1. Lentivirus production and viral transduction of 293T cells were performed as described above 8 h later, the medium was replaced with fresh medium. Virus-containing medium were collected after 48 h and 72 h transfection.

ChAT overexpression. Expression vectors for full-length of ChAT (pHS-AVC-LW2172) and corresponding empty vector (pHS-BVC-LW233) were purchased from Syngentech. Expression vectors for ChAT-212 short isoform (pHS-AVC-1609) and corresponding empty vector (pHS-B-0165) were purchased from Syngentech. All vectors were employed to construct the lentivirus according to previously described. PC-9 and HCC827 cells were transduced with lentivirus.

Generation of ChAT CRISPR/Cas9 knockout in PC9 cells. plentiCRISPR v2-ChAT sgRNA (pHS-ACR-0205/pHS-ACR-0206) and corresponding empty vector (pHS-ACR-LW1035) were purchased from Syngentech. All vectors were confirmed by DNA sequencing. Lentivirus were

produced also according to previously described. PC-9 cells were transduced with lentivirus for 72 h and then selected with puromycin. Selection of monoclonal cells were performed.

Western blot analysis. Protein lysates were prepared in RIPA buffer and quantified using the BCA protein assay kit (Beyotime). Proteins were separated on SDS-PAGE gels, transferred to PVDF membranes, and probed with antibodies against ChAT (1:1000, Proteintech, 20747-1-AP), GAPDH (1:3000, Proteintech, 60004-1-lg), α-Tubulin (1:3000, Proteintech, 11224-1-AP), HDAC3 (1:1000, Proteintech, 10255-1-AP), β-catenin (1:1000, Cell Signaling, 8480), Non-phospho (Active) β-catenin (1:1000, Cell Signaling, 8814), AChE (1:1000, Abcam, ab31276), VAChT (1:1000, Abcam, ab235201), M3R (1:1000, Abcam, ab126168), CHT1 (1:1000, Proteintech, 21848-1-AP), YAP (1:1000, ABclonal, A1002), FLAG M2 (1:1000, Sigma-Aldrich, F1804). After overnight incubation with primary antibody at 4°C, washing, and incubation with secondary antibodies, blots were performed using a chemiluminescence system (Pierce). To detect proteins with similar size, we did membrane stripping for re-probing.

Immunohistochemistry. For histology, samples were formalin-fixed, paraffin-embedded, sectioned at 5 μm and stained with H&E according to standard histopathological techniques. For immunohistochemical analysis, 5 μm paraffin sections were deparaffinized and rehydrated. The endogenous peroxidase was inactivated with a 3% hydrogen peroxide/methanol solution (ZSGB-BIO, PV-9001, reagent 1) and washed with PBS. After incubation for 15 min in a boiling water bath for antigen retrieval. Block in 10% normal serum with 1% BSA in TBS for 2 h at room temperature. Sections were incubated with primary antibodies diluted in blocking buffer overnight at 4°C. Primary antibodies used were the following: ChAT (1:100, Merck, AB143), WNT6 (1:25, Abcam, ab150588), WNT9A (1:50, Abcam, Ab125957), AChE (1:400, Abcam, Ab97299), M3R (1:100, Abcam, Ab87199), VAChT (1:50, Abcam, Ab235201), Ki-67 (1:200,

Abcam, Ab15580), pEGFR (1:50, CST, 3777), pERK (1:200, CST, 4370), pAKT (1:100, CST, 4060). The sections were washed with PBS for 3 times, and then incubated with antibody enhancing solution (ZSGB-BIO, PV-9001, reagent 2) for 20 min. The sections were washed with PBS for 3 times and then incubated with secondary antibody (ZSGB-BIO, PV-9001, reagent 3) for 20 min at room temperature. Samples were visualized with a scanning microscope (Pannoramic SCAN) and images were acquired in a bright field using the GS3-U3-51S5M-C system and processed using Case Viewer software (Pannoramic SCAN). Brightness and contrast for representative images were adjusted equally among groups. Images were captured using Case Viewer and immunoreactivity was evaluated with IHC Profiler as an image J plug-in in blinded manner. The evaluation was based on staining intensity and the extent of staining. Randomly selected fields were quantified cells staining positive values.

Generation of patient-derived xenografts. PDX-NSCLC645 and PDX-NSCLC868, derived from *EGFR*-mutant NSCLC patients, were conducted by LIDI Biotech Co. and CrownBio Co., respectively. The companies were responsible for any necessary IRB approvals and patient consents to allow the tumor tissue to be used in research. NSCLC645 was established from a 62-year-old woman with lung cancer containing exon *EGFR* 19 deletion & T790M and NSCLC868 was established from a woman with lung cancer containing *EGFR* L858R mutation & T790M. Tumor specimens were subcutaneously propagated in BALB/c nude mice. When tumors reached nearly 300 mm^3 in volume, 5 mg/kg Osimertinib was administered daily by oral gavage. Tumor size was evaluated twice a week using a caliper, and the volume of the mass was calculated using the formula $0.5 \times L \times W^2$, where L is the long diameter and W is the short diameter. To test the efficacy of the combination therapy in vitro, tumor cells derived from NSCLC645 were seeded

into 96-well plate and incubated with indicated drugs for 6 days. Cell viability was assessed using CellTiter Glo Luminescent Cell Viability assay.

Luciferase assay. Genomic region harboring ChAT enhancers（#1 and #3） was amplified by High-Fidelity PCR（NEB, M0492S）and cloned into pGL4.23[luc2-minP] vector (Miaolingbio, P0696). Briefly, ChAT enhancer reporter, control vector and Renilla (Addgene, 87121) were transfected into PC9 cells with shControl or shYAP using PEI transfection reagent (Proteintech, PR40001). Fresh media was replaced either with DMSO or with 2 μM Osimertinib post 12 h transfection. Cells were lysed 72 h later and detected for luciferase activity using the Dual-Luciferase Reporter Assay System (Promega, E1910). The firefly luciferase activity was normalized to Renilla.

ChIP-qPCR assays. The ChIP assay was performed using the SimpleChIP® Plus Sonication Chromatin IP Kit (CST, 56383), following the manufactures instructions. PC9 cells were cultured on 15 cm plates and treated either with DMSO or with 2 μM Osimertinib for 48 h. Then, cells were collected for further analysis according to the provided protocol. The following antibodies were used for ChIP: YAP (CST, 14074) or IgG (supplied with the kit).

All primer sequences used for ChIP-qPCR assay are as following:

ChAT-enhancer #1-forward: AGGGAGTCTGGAATCTGCTC, reverse: AGCCATTCCTCATTTGAACATCT;

ChAT-enhancer #2-forward: TAGCCAAGCAGAAGACGGG, reverse: TCAGTGCAAGGAGACAGGTT;

ChAT-enhancer #3-forward: GGCATGGGTGTAGAGGACAT, reverse: CCTTCTTTTCCTAAGGCTGCTT;

Xenograft experiments. All animal protocols related to mouse experiments were approved by the University of Tsinghua Institutional Animal Care and Use Committee. The animal protocols are compliant with all relevant ethical regulations. Female BALB/c nude mice aged 6–8 weeks were purchased from Charles River Laboratories Inc., Wilmington, MA. For experiments testing the effects of ChAT on MRD formation and formed MRD. Wild type PC9 or PC9 ChAT CRISPR/Cas9 knockout cells (3×10^6 cells per mouse) were injected subcutaneously into the right flanks of mice. After tumor establishment, tumor-bearing mice with PC9 WT and ChAT knockout cells were all randomly assigned to receive either vehicle or Osimertinib (1 mg/kg/d), all agents were administered by oral gavage. Body weights were measured over the course of treatment, and tumor growth was monitored by caliper measurements twice weekly until endpoint was reached and mice were euthanized. In addition, PC9 cells (3×10^6 cells per mouse) with shChAT or control shRNA, and HCC827 cells (3×10^6 cells per mouse) with stably full-length ChAT overexpression or negative controls were injected subcutaneously into the right flanks of mice. After tumor establishment, mice were randomly placed into groups to receive either vehicle or Osimertinib. Mice were treated with drugs using doses for the indicated time described in the figure legends.

For the experiments testing the effects of Darifenacin on the MRD formation, PC9 cells (5×10^6 cells per mouse) were injected subcutaneously into the right flanks of mice. After tumor establishment, mice were randomly placed into different groups. Groups were treated as follows: Group 1: vehicle daily to the end of the study. Group 2: Darifenacin (5 mg/kg/d, intra-peritoneal injection) alone to the end of the study. Group 3: Osimertinib (5 mg/kg/d, oral gavage) alone for

9 days + vehicle from day 9 to the end of the study. Group 4: combination of Osimertinib (5 mg/kg/d, oral gavage) with Darifenacin (5 mg/kg/d, intra-peritoneal injection) for 9 days + vehicle from day 9 to the end of the study. Group 5: combination of Osimertinib (5 mg/kg/d, oral gavage) with Darifenacin (5 mg/kg/d, intra-peritoneal injection) for 9 days + Darifenacin (5 mg/kg/d, intra-peritoneal injection) from day 9 to the end of the study. For the experiment testing the effects of Darifenacin on the formed MRD, PC9 xenografts were treated with Osimertinib (1 mg/kg/d, oral gavage) for 9 days and randomly assigned to treatment with Osimertinib (1 mg/kg/d, oral gavage) alone or a combination of Darifenacin (5 mg/kg/d, intra-peritoneal injection).

For the experiments testing the functional role of ChAT short isoform in driving drug tolerance, the xenograft models were established by injection of PC9 cells with stable overexpression of ChAT short isoform or negative controls. PC9 cells with stable overexpression of ChAT short isoform or negative controls cells (3×10^6 cells per mouse) were injected subcutaneously into the right flanks of mice. After tumor establishment, mice were randomly placed into different groups as indicated. Xenografts mice were treated with vehicle or Osimertinib (5 mg/kg/d, oral gavage) for 9 days, followed by drug withdrawal.

For the experiments exploring whether exogenous ACh supplementation can rescue the growth defect caused by ChAT knockout, we pretreated PC9 WT and ChAT knockout cells with or without exogenous ACh for 3 days, which were injected subcutaneously into the right flanks of mice (3×10^6 cells per mouse). After tumor establishment, the mice were pretreated with ACh (20 mg/kg/d, subcutaneous injection) for 7 days and then were randomly placed into groups to receive corresponding vehicle, Osimertinib (5 mg/kg/d, oral gavage) alone, ACh (20 mg/kg/d,

subcutaneous injection) alone, or the combination of Osimertinib and ACh for 9 days, followed by Osimertinib withdrawal and continuous ACh treatment.

Tumor size was measured using a caliper and tumor volume was calculated using the standard formula: $0.5 \times L \times W^2$, where L is the long diameter and W is the short diameter. For survival analysis, Kaplan-Meier analysis was used for calculating percent survival using GraphPad Prism v.7.0. The percent survival was calculated when the tumors reached 1000 mm^3.

1. Sharma SV, Lee DY, Li B, Quinlan MP, Takahashi F, Maheswaran S, et al. A chromatin-mediated reversible drug-tolerant state in cancer cell subpopulations. *Cell.* 2010;141(1):69-80.
2. Agathocleous M, Meacham CE, Burgess RJ, Piskounova E, Zhao Z, Crane GM, et al. Ascorbate regulates haematopoietic stem cell function and leukaemogenesis. *Nature.* 2017;549(7673):476-81.
3. Huang F, Ni M, Chalishazar MD, Huffman KE, Kim J, Cai L, et al. Inosine Monophosphate Dehydrogenase Dependence in a Subset of Small Cell Lung Cancers. *Cell Metab.* 2018;28(3):369-82 e5.

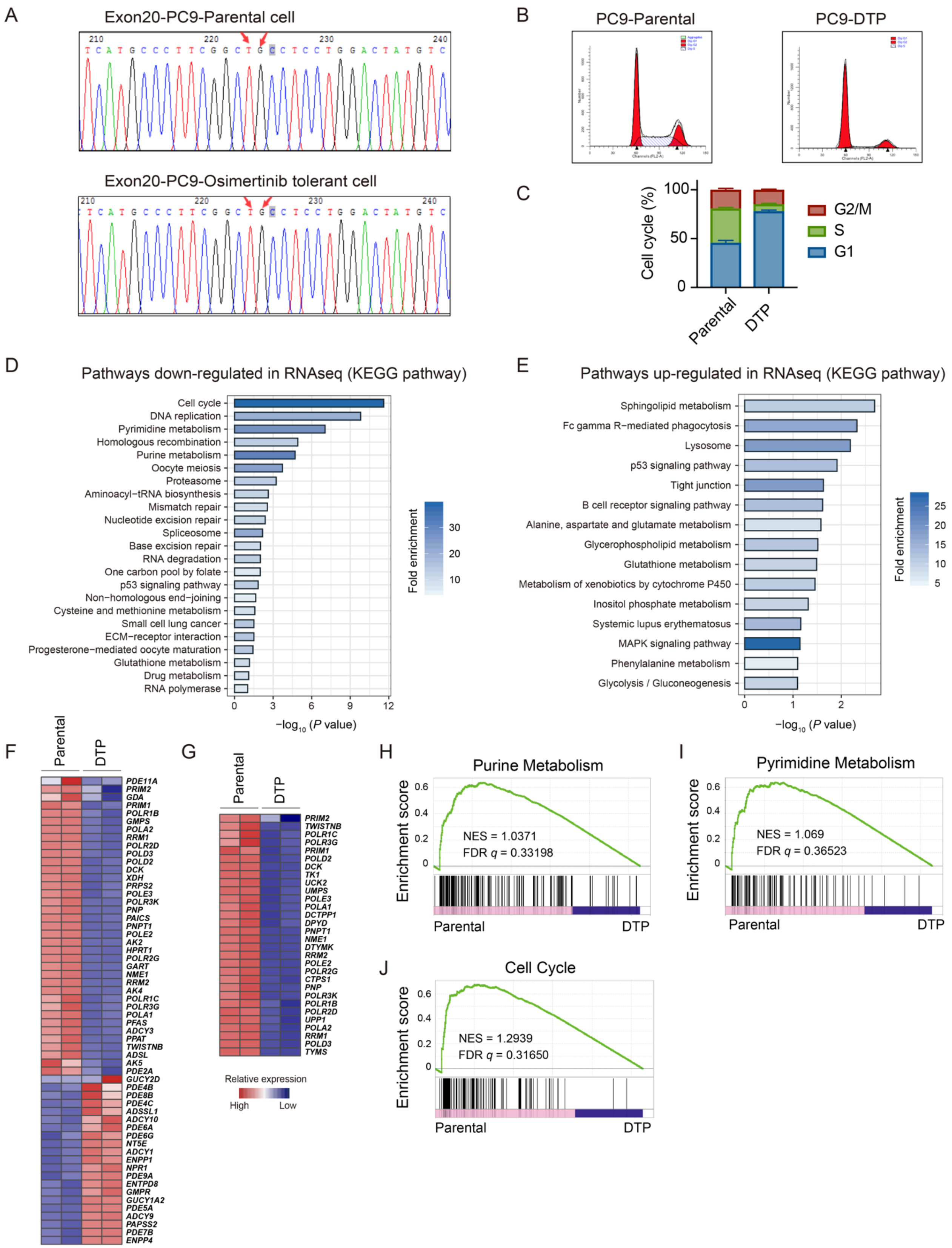
A
Exon20-PC9-Parental cell
Exon20-PC9-Osimertinib tolerant cell
B
PC9-Parental
PC9-DTP
C
Cell cycle (%)
G2/M
S
G1
Parental
DTP
D
Pathways down-regulated in RNAseq (KEGG pathway)
Cell cycle
DNA replication
Pyrimidine metabolism
Homologous recombination
Purine metabolism
Oocyte meiosis
Proteasome
Aminoacyl−tRNA biosynthesis
Mismatch repair
Nucleotide excision repair
Spliceosome
Base excision repair
RNA degradation
One carbon pool by folate
p53 signaling pathway
Non−homologous end−joining
Cysteine and methionine metabolism
Small cell lung cancer
ECM−receptor interaction
Progesterone−mediated oocyte maturation
Glutathione metabolism
Drug metabolism
RNA polymerase
Fold enrichment
−log10 (P value)
E
Pathways up-regulated in RNAseq (KEGG pathway)
Sphingolipid metabolism
Fc gamma R−mediated phagocytosis
Lysosome
p53 signaling pathway
Tight junction
B cell receptor signaling pathway
Alanine, aspartate and glutamate metabolism
Glycerophospholipid metabolism
Glutathione metabolism
Metabolism of xenobiotics by cytochrome P450
Inositol phosphate metabolism
Systemic lupus erythematosus
MAPK signaling pathway
Phenylalanine metabolism
Glycolysis / Gluconeogenesis
Fold enrichment
−log10 (P value)
F
Parental
DTP
G
Parental
DTP
Relative expression
High
Low
H
Purine Metabolism
Enrichment score
NES = 1.0371
FDR q = 0.33198
Parental
DTP
I
Pyrimidine Metabolism
Enrichment score
NES = 1.069
FDR q = 0.36523
Parental
DTP
J
Cell Cycle
Enrichment score
NES = 1.2939
FDR q = 0.31650
Parental
DTP

Supplemental Figure 1. Characterization of drug-tolerant persister cancer cells. (**A**) Analysis of EGFR exon 20 sequence variations of parental PC9 cell (top) and Osimertinib-tolerant PC9 cells (bottom). Repeated two times with similar results. (**B and C**) Distribution of parental PC9 and Osimertinib-tolerant PC9 cells in G1, S and G2 phases. (**D and E**) KEGG pathway enrichment for down-regulated genes and up-regulated genes identified by RNA-seq data of Osimertinib-tolerant PC9 cells compared to parental PC9 cells. (**F and G**) Heatmap of purine metabolism and pyrimidine metabolism related gene expression value in Osimertinib-tolerant PC9 cells compared to parental PC9 cells. Rows are z-scores calculated for each gene in both cell types. (**H-J**) Gene enrichment analysis of RNA-seq data using gene set enrichment analysis (GSEA). Normalized enrichment score (NES) and false-discovery rate (FDR) q value are shown.

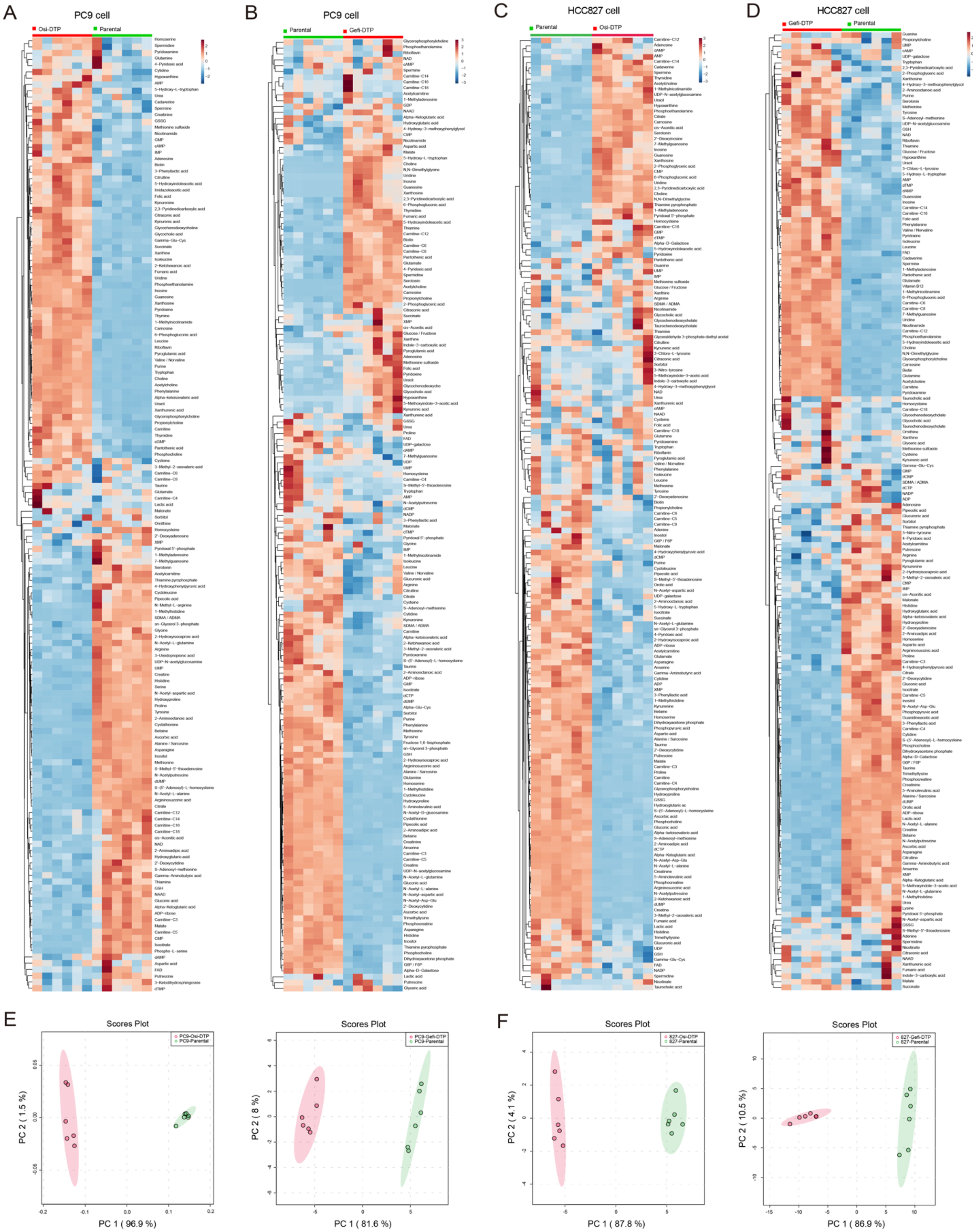
A
PC9 cell
Osi-DTP
Parental
B
PC9 cell
Parental
Gefi-DTP
C
HCC827 cell
Parental
Osi-DTP
D
HCC827 cell
Gefi-DTP
Parental
E
Scores Plot
PC9-Osi-DTP
PC9-Parental
PC 1 (96.9 %)
PC 2 (1.5 %)
Scores Plot
PC9-Gefi-DTP
PC9-Parental
PC 1 (81.6 %)
PC 2 (8 %)
F
Scores Plot
827-Osi-DTP
827-Parental
PC 1 (87.8 %)
PC 2 (4.1 %)
Scores Plot
827-Gefi-DTP
827-Parental
PC 1 (86.9 %)
PC 2 (10.5 %)

Supplemental Figure 2. Metabolomics analysis of the EGFR-TKI induced DTP cells. (**A-D**) Relative abundance of metabolites extracted from Osimertinib-induced DTP cells, Gefitinib-induced DTP cells and parental cells of PC9 and HCC827. Peak area of each metabolite was normalized by the sum of peak areas of all detected metabolites in that sample and the heatmap displays the normalized data for each metabolite (n = 6). (**E**) Principal-component analysis of the metabolite detected from parental PC9 cells, Osimertinib-induced DTP cells (left) and Gefitinib-induced DTP cells (right) (n = 6). (**F**) Principal-component analysis of the metabolites detected from parental HCC827 cells, Osimertinib-induced DTP cells (left) and Gefitinib-induced DTP cells (right) (n = 6).

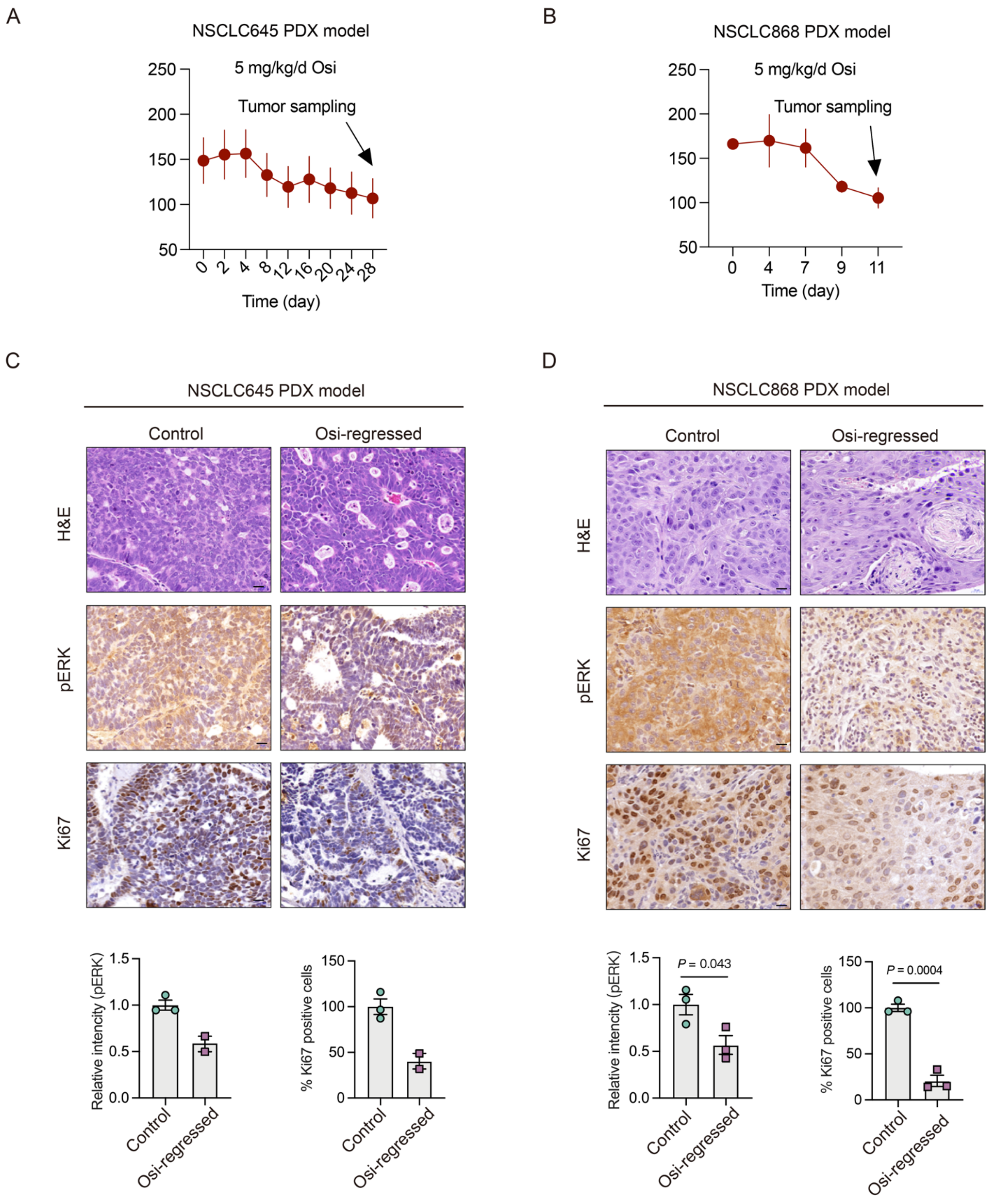

Supplemental Figure 3. Establishment of the Osi-regressed tumors in NSCLC PDX models.

(**A and B**) Tumor volume of NSCLC645 PDX model and NSCLC868 PDX model exposed to 5

mg/kg/d Osimertinib (n = 3). (**C and D**) Representative image and quantification of immunohistochemical staining of Ki67 and pERK on the tumor sections from control and Osi-regressed tumors from NSCLC645 PDX model and NSCLC868 PDX model (n = 2 or 3). Scale bar, 20 μm. Data are shown as mean ± s.e.m., two-tailed Student's t test was used.

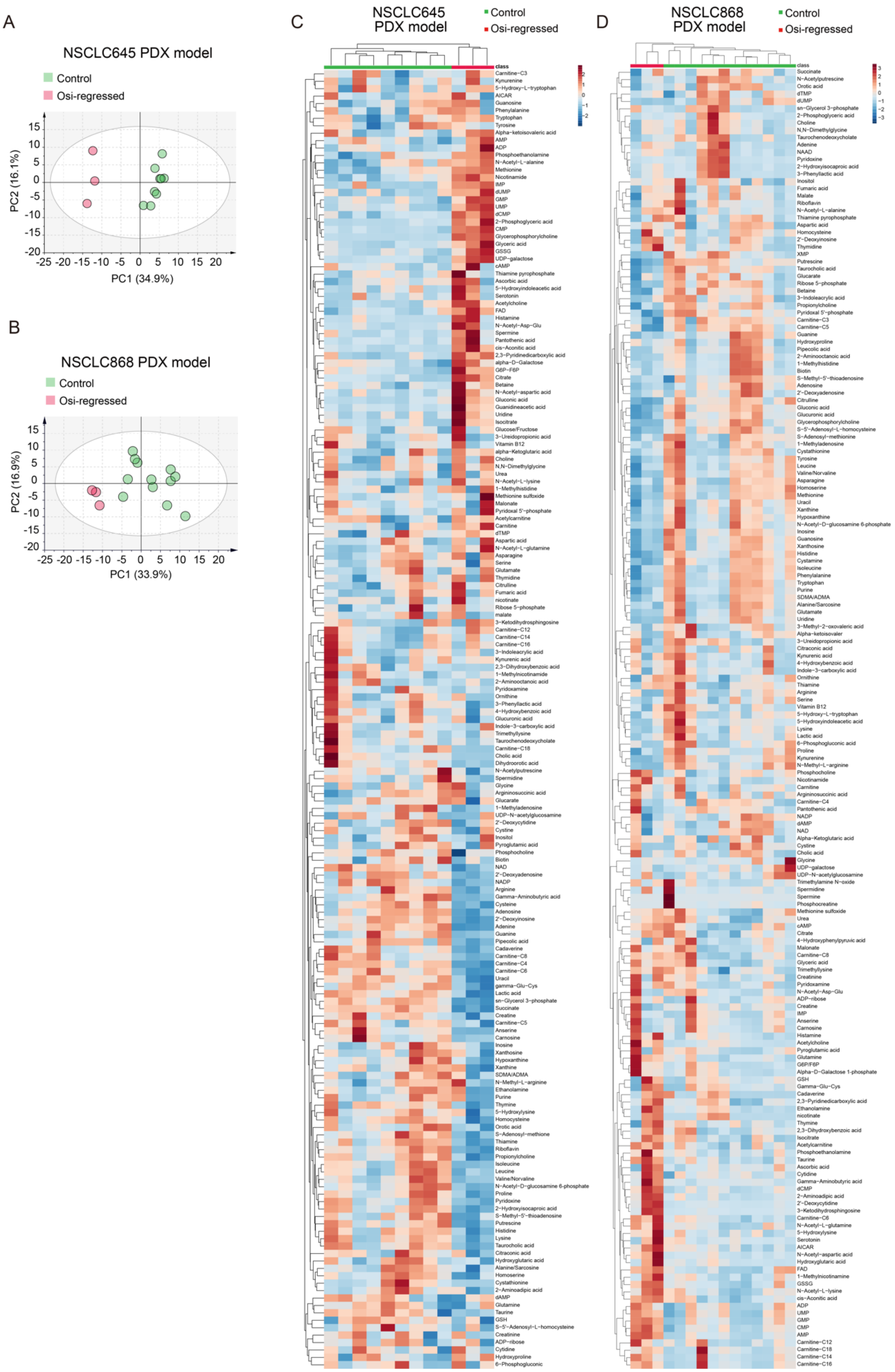
A
NSCLC645 PDX model
Control
Osi-regressed
PC1 (34.9%)
PC2 (16.1%)
B
NSCLC868 PDX model
Control
Osi-regressed
PC1 (33.9%)
PC2 (16.9%)
C
NSCLC645 PDX model
Control
Osi-regressed
D
NSCLC868 PDX model
Control
Osi-regressed

Supplemental Figure 4. Metabolomics analysis of the Osi-regressed tumors in NSCLC PDX models. (**A and B**) Principal-component analysis of the metabolites detected from NSCLC645 PDX model and NSCLC868 PDX model. (**C and D**) Relative abundance of metabolites extracted from control and Osi-regressed tumors from the NSCLC645 PDX model and the NSCLC868 PDX model. Peak area of each metabolite was normalized by the sum of peak areas of all detected metabolites in that sample and the heatmap displays the normalized data for each metabolite. (**A-D**) n = 3 or 4 mice, three fragments were harvested for analysis in each tumor of controls.

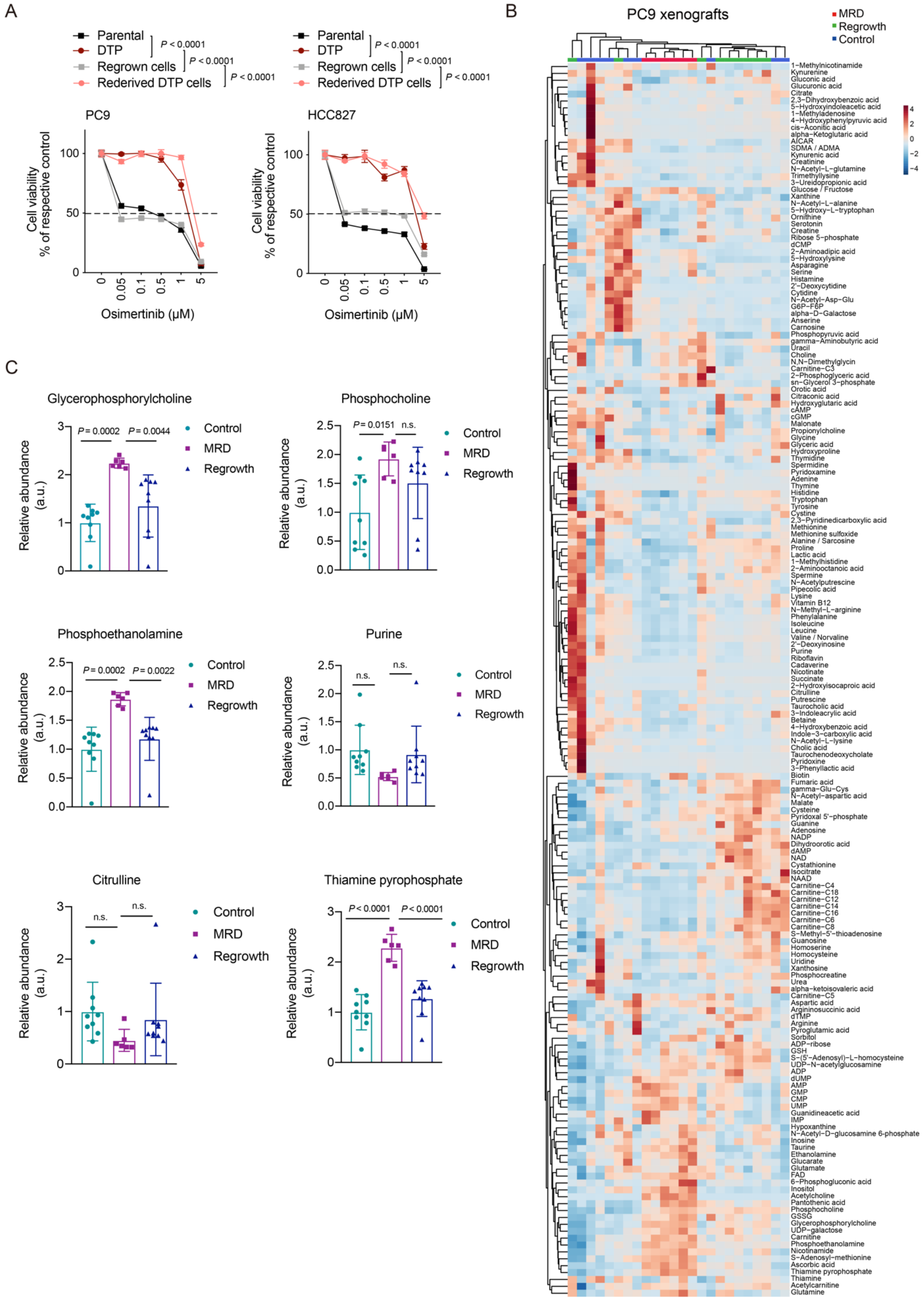
A
Parental
DTP
Regrown cells
Rederived DTP cells
P < 0.0001
P < 0.0001
P < 0.0001
PC9
HCC827
Cell viability
% of respective control
100
50
0
0
0.05
0.1
0.5
1
5
Osimertinib (μM)
B
PC9 xenografts
MRD
Regrowth
Control
4
2
0
−2
−4
1−Methylnicotinamide
Kynurenine
Gluconic acid
Glucuronic acid
Citrate
2,3−Dihydroxybenzoic acid
5−Hydroxyindoleacetic acid
1−Methyladenosine
4−Hydroxyphenylpyruvic acid
cis−Aconitic acid
alpha−Ketoglutaric acid
AICAR
SDMA / ADMA
Kynurenic acid
Creatinine
N−Acetyl−L−glutamine
Trimethyllysine
3−Ureidopropionic acid
Glucose / Fructose
Xanthine
N−Acetyl−L−alanine
5−Hydroxy−L−tryptophan
Ornithine
Serotonin
Creatine
Ribose 5−phosphate
dCMP
2−Aminoadipic acid
5−Hydroxylysine
Asparagine
Serine
Histamine
2'−Deoxycytidine
Cytidine
N−Acetyl−Asp−Glu
G6P−F6P
alpha−D−Galactose
Anserine
Carnosine
Phosphopyruvic acid
gamma−Aminobutyric acid
Uracil
Choline
N,N−Dimethylglycin
Carnitine−C3
2−Phosphoglyceric acid
sn−Glycerol 3−phosphate
Orotic acid
Citraconic acid
Hydroxyglutaric acid
cAMP
cGMP
Malonate
Propionylcholine
Glycine
Glyceric acid
Hydroxyproline
Thymidine
Spermidine
Pyridoxamine
Adenine
Thymine
Histidine
Tryptophan
Tyrosine
Cystine
2,3−Pyridinedicarboxylic acid
Methionine
Methionine sulfoxide
Alanine / Sarcosine
Proline
Lactic acid
1−Methylhistidine
2−Aminooctanoic acid
Spermine
N−Acetylputrescine
Pipecolic acid
Lysine
Vitamin B12
N−Methyl−L−arginine
Phenylalanine
Isoleucine
Leucine
Valine / Norvaline
2'−Deoxyinosine
Purine
Riboflavin
Cadaverine
Nicotinate
Succinate
2−Hydroxyisocaproic acid
Citrulline
Putrescine
Taurocholic acid
3−Indoleacrylic acid
Betaine
4−Hydroxybenzoic acid
Indole−3−carboxylic acid
N−Acetyl−L−lysine
Cholic acid
Taurochenodeoxycholate
Pyridoxine
3−Phenyllactic acid
Biotin
Fumaric acid
gamma−Glu−Cys
N−Acetyl−aspartic acid
Malate
Cysteine
Pyridoxal 5'−phosphate
Guanine
Adenosine
NADP
Dihydroorotic acid
dAMP
NAD
Cystathionine
Isocitrate
NAAD
Carnitine−C4
Carnitine−C18
Carnitine−C12
Carnitine−C14
Carnitine−C16
Carnitine−C6
Carnitine−C8
S−Methyl−5'−thioadenosine
Guanosine
Homoserine
Homocysteine
Uridine
Xanthosine
Phosphocreatine
Urea
alpha−ketoisovaleric acid
Carnitine−C5
Aspartic acid
Argininosuccinic acid
dTMP
Arginine
Pyroglutamic acid
Sorbitol
ADP−ribose
GSH
S−(5'−Adenosyl)−L−homocysteine
UDP−N−acetylglucosamine
ADP
dUMP
AMP
GMP
CMP
UMP
Guanidineacetic acid
IMP
Hypoxanthine
N−Acetyl−D−glucosamine 6-phosphate
Inosine
Taurine
Ethanolamine
Glucarate
Glutamate
FAD
6−Phosphogluconic acid
Inositol
Acetylcholine
Pantothenic acid
Phosphocholine
GSSG
Glycerophosphorylcholine
UDP−galactose
Carnitine
Phosphoethanolamine
Nicotinamide
S−Adenosyl−methionine
Ascorbic acid
Thiamine pyrophosphate
Thiamine
Acetylcarnitine
Glutamine
C
Glycerophosphorylcholine
P = 0.0002
P = 0.0044
Phosphocholine
P = 0.0151
n.s.
Phosphoethanolamine
P = 0.0002
P = 0.0022
Purine
n.s.
n.s.
Citrulline
n.s.
n.s.
Thiamine pyrophosphate
P < 0.0001
P < 0.0001
Control
MRD
Regrowth
Relative abundance
(a.u.)
0
1
2
3
0.0
0.5
1.0
1.5
2.0
2.5

Supplemental Figure 5. Metabolomics analysis of the MRD and regrown tumors after drug withdrawal of PC9 xenografts. (**A**) Dose-response curves of PC9 or HCC827 parental, DTP, regrown and re-derived DTP cells incubated in increasing concentrations of Osimertinib for 72 h (*n* = 5). (**B**) Relative abundance of metabolites extracted from control (*n* = 9), MRD (*n* = 6) and regrown tumors (*n* = 9) from PC9 xenograft mouse models. Peak area of each metabolite was normalized by the sum of peak areas of all detected metabolites in that sample and the heatmap displays the normalized data for each metabolite. (**C**) Alterations of metabolites associated with choline metabolism. (**A and C**) Data are shown as mean ± s.e.m., two-way ANOVA adjusted by Bonferroni's correction (**A**) and one-way ANOVA with Tukey's test (**C**) was used.

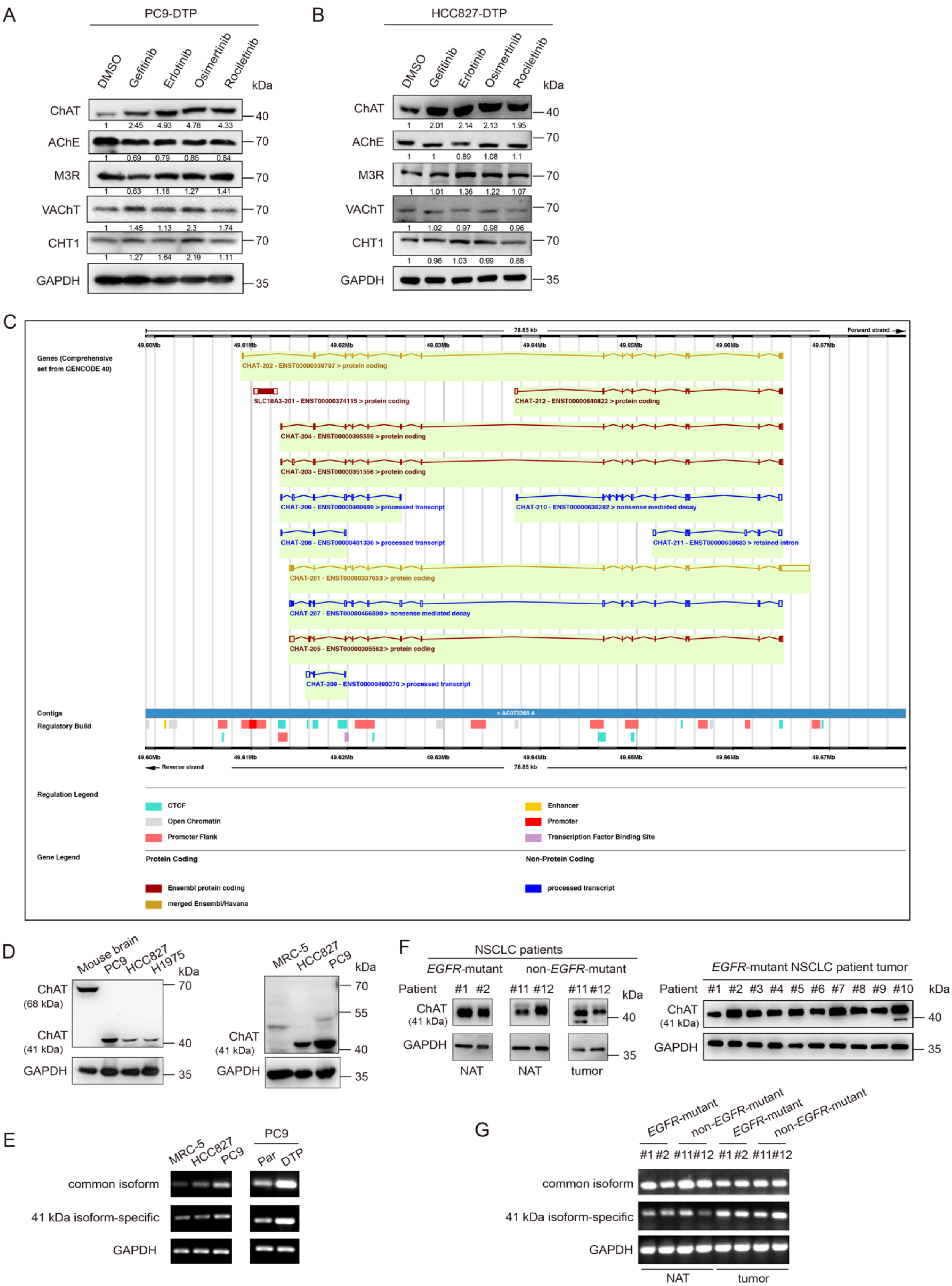

A
PC9-DTP
DMSO
Gefitinib
Erlotinib
Osimertinib
Rociletinib
kDa
ChAT
AChE
M3R
VAChT
CHT1
GAPDH
B
HCC827-DTP
C
Contigs
Regulatory Build
Regulation Legend
CTCF
Open Chromatin
Promoter Flank
Enhancer
Promoter
Transcription Factor Binding Site
Gene Legend
Protein Coding
Non-Protein Coding
Ensembl protein coding
merged Ensembl/Havana
processed transcript
D
Mouse brain
PC9
HCC827
H1975
MRC-5
ChAT (68 kDa)
ChAT (41 kDa)
F
NSCLC patients
EGFR-mutant
non-EGFR-mutant
Patient
NAT
tumor
EGFR-mutant NSCLC patient tumor
E
common isoform
41 kDa isoform-specific
Par
DTP
G

Supplemental Figure 6. The expression of key proteins associated with ACh metabolism and signaling. (**A and B**) Western blot analysis of indicated proteins from PC9 and HCC827 DTP cells induced by 2 μM indicated drugs for 9 days or in the parental cells. (**C**) Different transcripts of ChAT gene via alternative splicing were explored by Ensembl database. (**D**) Western blot analysis of ChAT isoforms in mouse brain, *EGFR*-mutant NSCLC cell lines and normal lung fibroblast cell line. (**E**) RT-PCR of ChAT isoforms in *EGFR*-mutant NSCLC cell lines and normal lung fibroblast cell line. (**F**) Western blot analysis of short ChAT isoform (41 kDa) in tumor and normal adjacent tissues of NSCLC patients with or without *EGFR* mutation. (**G**) RT-PCR of ChAT isoforms in tumor and normal adjacent tissues of NSCLC patients with or without *EGFR* mutation.

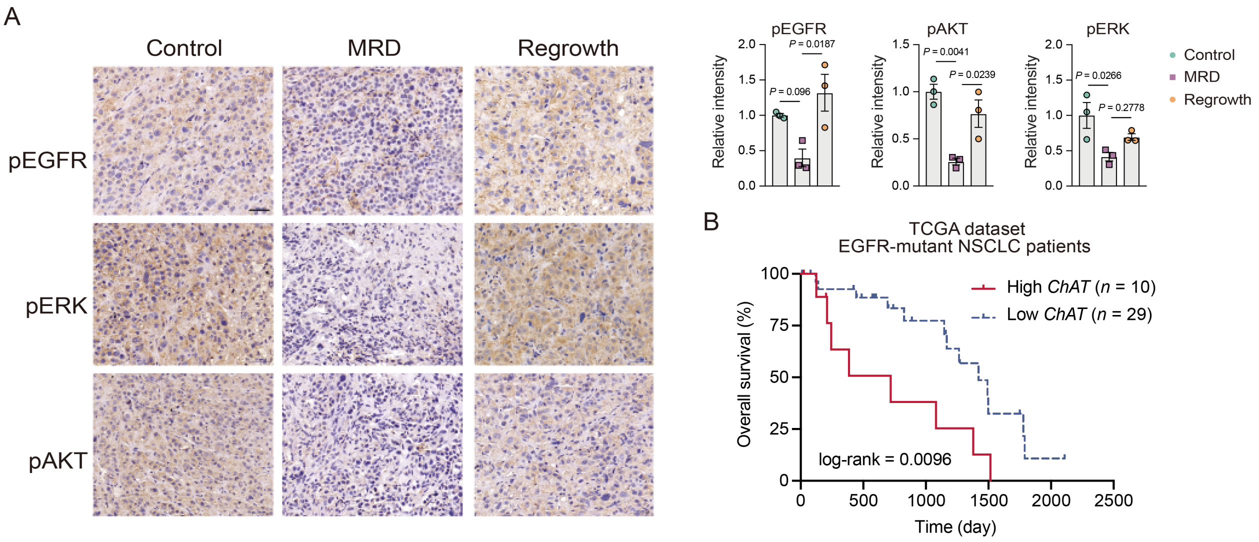

Supplemental Figure 7. Immunohistochemistry analysis of PC9 xenograft mouse models. (**A**) Representative image and quantification of immunohistochemical staining of pEGFR, pAKT, pERK on the tumor sections from control, MRD and regrown tumors from PC9 xenograft mouse models. Scale bar, 50 µm. Data are shown as mean ± s.e.m., *n* = 3 mice per group, one-way ANOVA with Tukey's test was used. (**B**) Kaplan-Meier curve showing the correlation between ChAT expression and overall survival. RNA-seq data and clinical information of *EGFR*-mutant NSCLC patients of TCGA dataset were obtained from the cbioportal website. Low expression of ChAT was defined as z-score ≤ -1. Log-rank test was used.

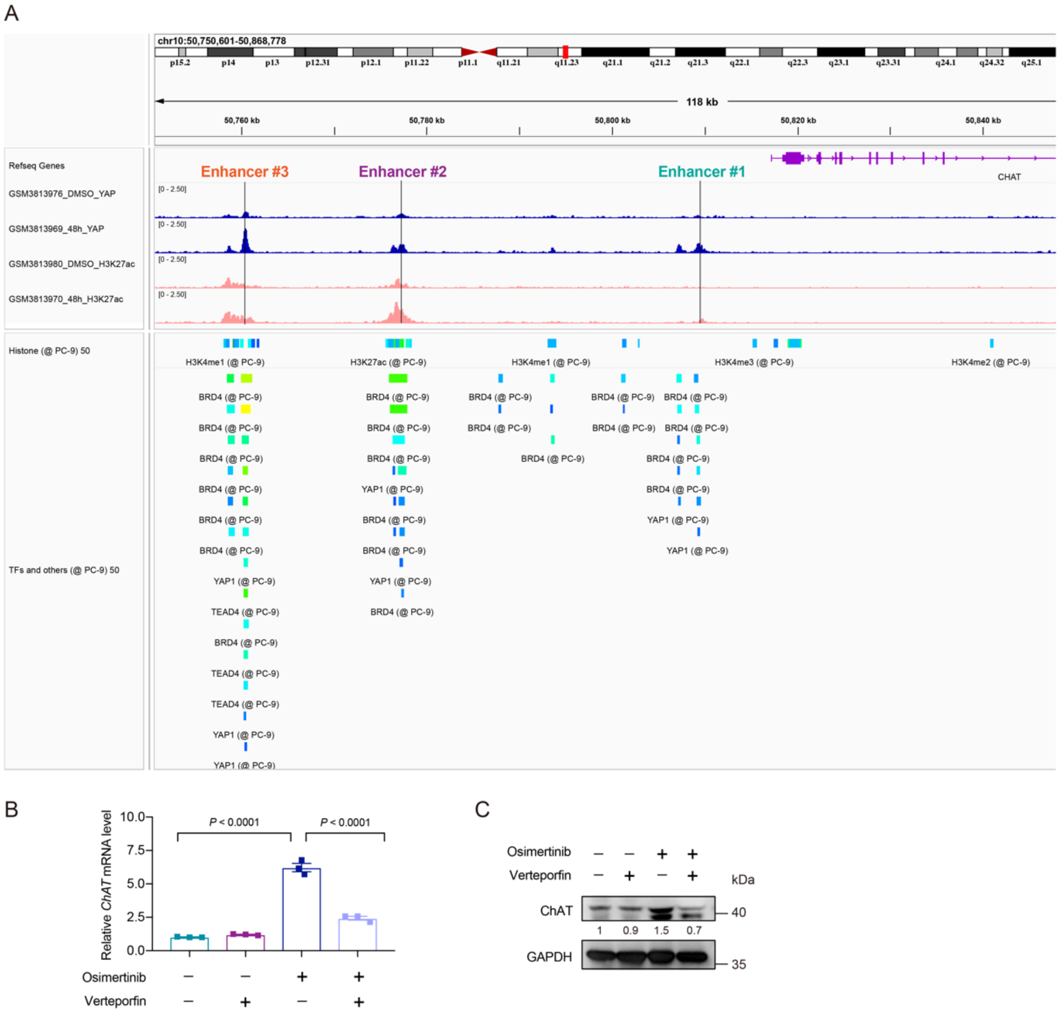

Supplemental Figure 8. The binding site of YAP/TEAD complex is located around the potential enhancer regions of ChAT gene. (**A**) Visualization of YAP/TEAD ChIP-seq signal around the potential enhancers of ChAT gene in PC9 cells from the public ChIP-seq data integrated by ChIP-Atlas. ChIP-seq data published in previous study (GSE131687) show increased ChIP-seq signal of YAP and H3K27ac around three potential enhancers (#1, #2 and #3) of ChAT gene in PC9 cells treated with the combination of 100 nM Osimertinib and 30 nM trametinib for 48 h compared to the cells treated with DMSO. (**B and C**) Relative mRNA and protein level of ChAT in PC9 cells treated with 2 μM Osimertinib with or without 2 μM

Verteporfin for 6 days (*n* = 3). Data are shown as mean ± s.e.m., one-way ANOVA with Tukey's test.

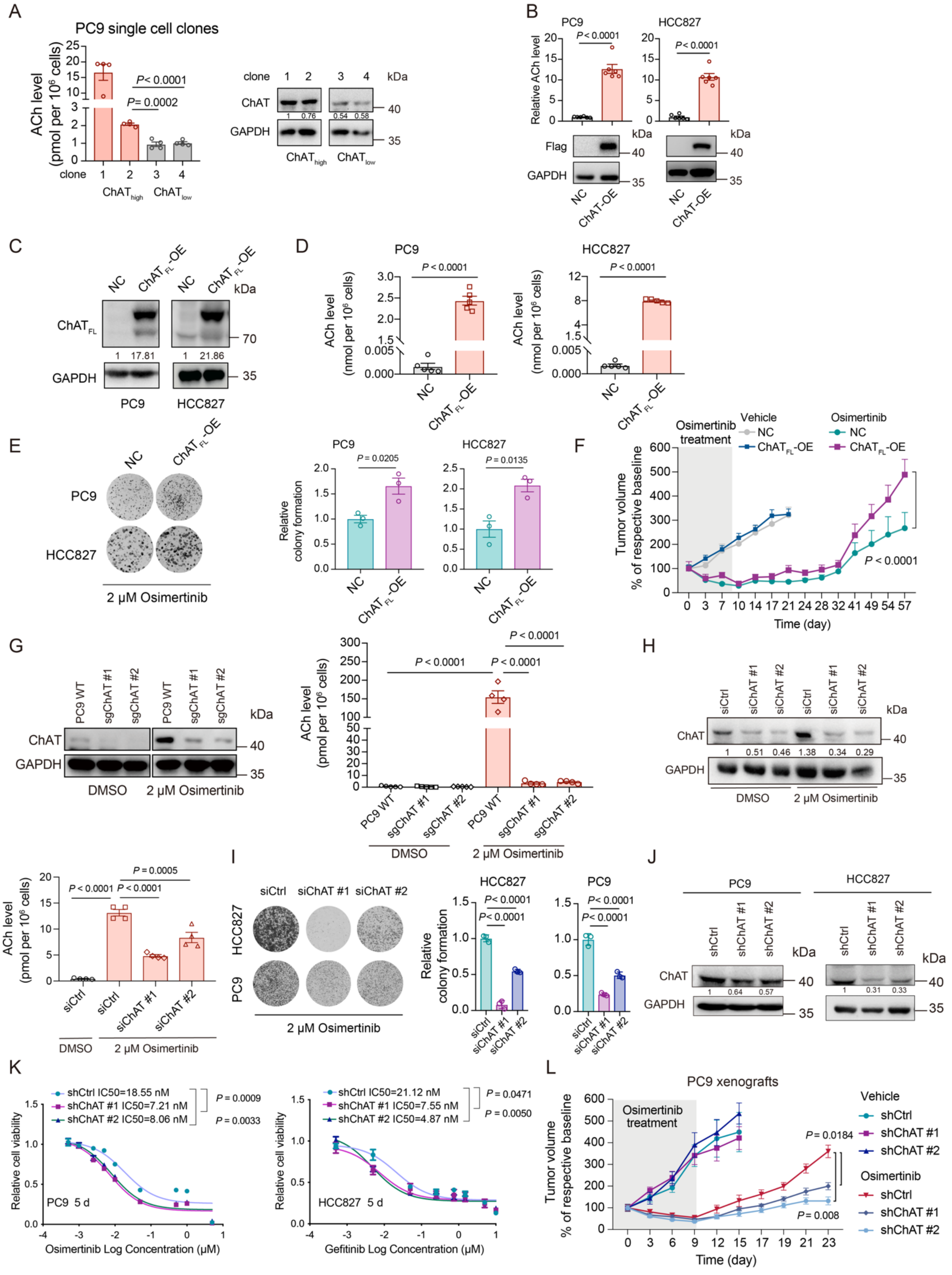
A
PC9 single cell clones
ACh level (pmol per 10^6 cells)
P< 0.0001
P= 0.0002
clone 1 2 3 4
ChAT high ChAT low
ChAT
GAPDH
kDa
B
PC9
HCC827
Relative ACh level
P< 0.0001
Flag
GAPDH
NC
ChAT-OE
C
NC
ChAT FL -OE
kDa
PC9
HCC827
D
ACh level (nmol per 10^6 cells)
E
2 μM Osimertinib
Relative colony formation
P = 0.0205
P = 0.0135
F
Osimertinib treatment
Vehicle
Osimertinib
NC
Tumor volume % of respective baseline
P < 0.0001
Time (day)
G
PC9 WT
sgChAT #1
sgChAT #2
DMSO
2 μM Osimertinib
H
siCtrl
siChAT #1
siChAT #2
I
J
K
shCtrl IC50=18.55 nM
shChAT #1 IC50=7.21 nM
shChAT #2 IC50=8.06 nM
P = 0.0009
P = 0.0033
Relative cell viability
PC9 5 d
Osimertinib Log Concentration (μM)
shCtrl IC50=21.12 nM
shChAT #1 IC50=7.55 nM
shChAT #2 IC50=4.87 nM
P = 0.0471
P = 0.0050
HCC827 5 d
Gefitinib Log Concentration (μM)
L
PC9 xenografts
Osimertinib treatment
Vehicle
shCtrl
shChAT #1
shChAT #2
Osimertinib
P = 0.0184
P = 0.008
Time (day)

Supplemental Figure 9. The role of ChAT in modulating DTP cells formation and residual tumor relapse. (**A**) ACh levels and short ChAT isoform expression in PC9 single cell clones (*n* = 4). (**B**) Relative ACh levels and short ChAT isoform expression in cells with Flag-ChAT short isoform overexpression (ChAT-OE, 41 kDa) and negative control (NC) (*n* = 6). (**C**) Western blot showing the overexpression of full-length ChAT. (**D**) Quantification of ACh levels in cells with overexpression of full-length ChAT ($ChAT_{FL}$-OE) or negative control (NC) (*n* = 5). (**E**) Colony formation assay of cells with $ChAT_{FL}$-OE or NC exposed to the 2 μM Osimertinib for 9 days (*n* = 3). (**F**) HCC827 xenograft mice injected with $ChAT_{FL}$-OE or NC cells were treated with 5 mg/kg Osimertinib for 9 days, or treated with vehicle. *n* = 5 or 8 per group. (**G**) Quantification of ACh levels in PC9 wild type (WT) and ChAT knockout cells with or without 2 μM Osimertinib for 6 days (*n* = 4 or 5). ChAT sgRNA was confirmed by western blot in left. (**H**) Quantification of ACh levels in PC9 cells transfected with ChAT or control siRNA for 24 h and followed by 2 μM Osimertinib for 4 days (*n* = 4). ChAT siRNA was confirmed by western blot. (**I**) Colony formation assay of cells transfected with ChAT or control siRNA for 24 h and followed by 2 μM Osimertinib for 9 days (*n* = 3). (**J**) Western blot analysis of indicated proteins in cells with stably expressed control (shCtrl) and ChAT (shChAT) shRNAs. (**K**) Dose-response curves to Osimertinib in PC9 cells and Gefitinib in HCC827 cells expressing shCtrl or shChAT for 5 days (*n* = 5-6). IC50 values were compared with one-way ANOVA. (**L**) PC9 xenograft mice injected with ChAT or control shRNA cells were treated with vehicle or 5 mg/kg Osimertinib for 9 days. *n* = 5 or 6 per group. (**A and B, D-I, K and L**) Data are shown as mean ± s.e.m., two-tailed Student's *t* test (**B, D and E**), one-way ANOVA with Tukey's test (**A, G and H**), one-way ANOVA with Dunnett's test (**I**), two-way ANOVA adjusted by Bonferroni's correction (**F and L**) were used.

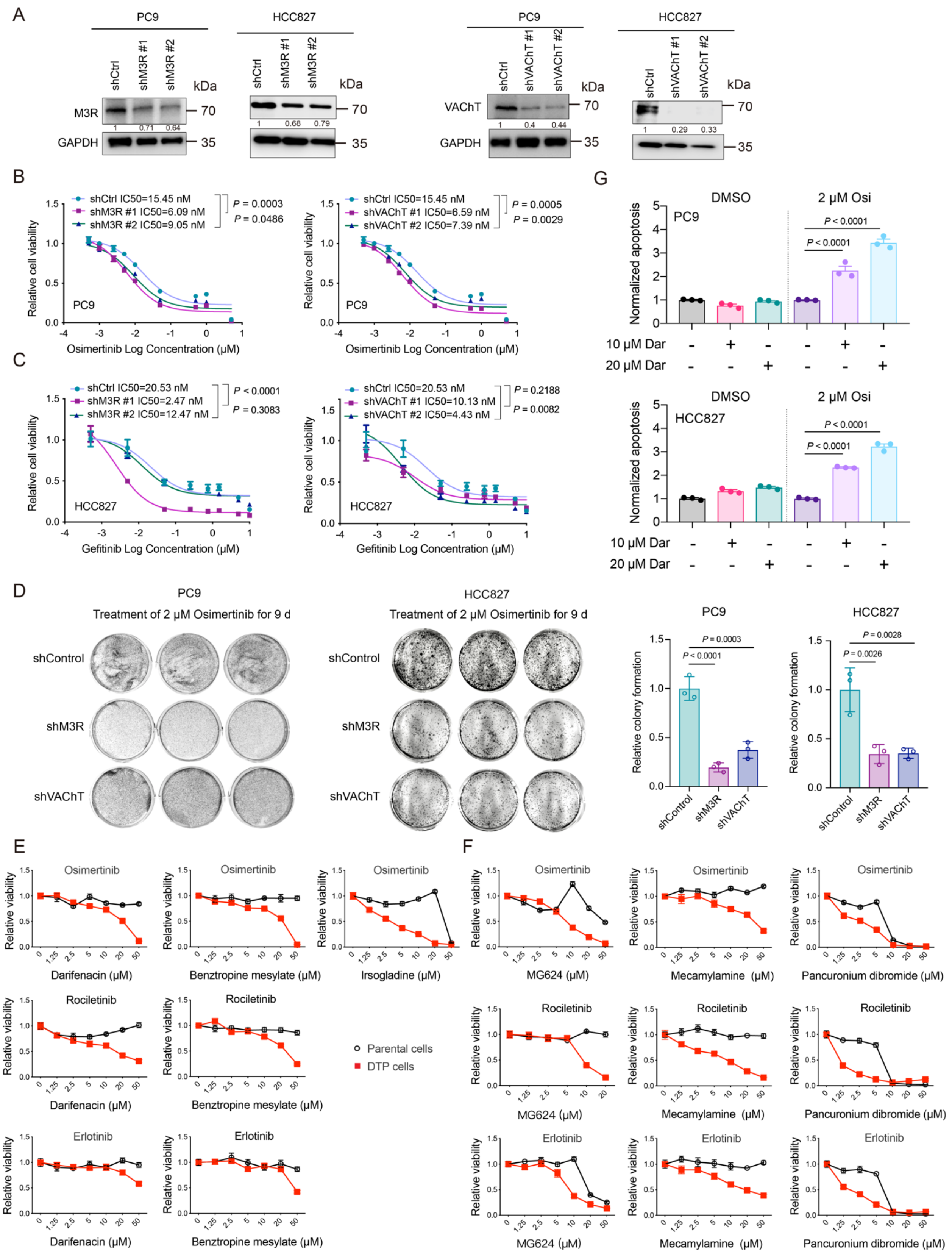
A
PC9
HCC827
shCtrl
shM3R #1
shM3R #2
kDa
M3R
GAPDH
70
35
1 0.71 0.64
1 0.68 0.79
VAChT
shVAChT #1
shVAChT #2
1 0.4 0.44
1 0.29 0.33
B
shCtrl IC50=15.45 nM
shM3R #1 IC50=6.09 nM
shM3R #2 IC50=9.05 nM
P = 0.0003
P = 0.0486
shVAChT #1 IC50=6.59 nM
shVAChT #2 IC50=7.39 nM
P = 0.0005
P = 0.0029
Relative cell viability
Osimertinib Log Concentration (μM)
C
shCtrl IC50=20.53 nM
shM3R #1 IC50=2.47 nM
shM3R #2 IC50=12.47 nM
P < 0.0001
P = 0.3083
shVAChT #1 IC50=10.13 nM
shVAChT #2 IC50=4.43 nM
P = 0.2188
P = 0.0082
Gefitinib Log Concentration (μM)
G
DMSO
2 μM Osi
Normalized apoptosis
P < 0.0001
10 μM Dar
20 μM Dar
D
Treatment of 2 μM Osimertinib for 9 d
shControl
shM3R
shVAChT
Relative colony formation
P = 0.0003
P < 0.0001
P = 0.0028
P = 0.0026
E
Osimertinib
Rociletinib
Erlotinib
Relative viability
Darifenacin (μM)
Benztropine mesylate (μM)
Irsogladine (μM)
Parental cells
DTP cells
F
MG624 (μM)
Mecamylamine (μM)
Pancuronium dibromide (μM)

Supplemental Figure 10. Blockade of ACh metabolism and signaling enhanced EGFR-TKI sensitivity of cancer cells and inhibited DTP formation. (**A**) Western blot analysis of indicated proteins in PC9 and HCC827 cells with stably expressed control (shCtrl), M3R (shM3R) or VAChT (shVAChT) shRNAs. (**B**) Dose-response curves to Osimertinib in PC9 cells expressing shCtrl, shM3R or shVAChT for 5 days. (**C**) Dose-response curves to Gefitinib in HCC827 cells expressing shCtrl, shM3R or shVAChT for 5 days. For **B** and **C**, IC50 values were compared with one-way ANOVA (n = 4-6). (**D**) Colony-formation assays were performed in PC9 and HCC827 cells with stably expressed shCtrl, shM3R or shVAChT and cultured with 2 μM Osimertinib for 9 days (n = 3). (**E and F**) Dose-response curves of PC9 parental, Osimertinib-tolerant, Rociletinib-tolerant and Erlotinib-tolerant cells incubated in increasing concentrations of indicated compounds for 6 days (n = 6). (**G**) Normalized apoptosis of PC9 and HCC827 cells treated with indicated drugs for 6 days (n = 3). (**B-G**) Data are shown as mean ± s.e.m., one-way ANOVA with Dunnett's test (**D**) and one-way ANOVA with Tukey's test (**G**) was used.

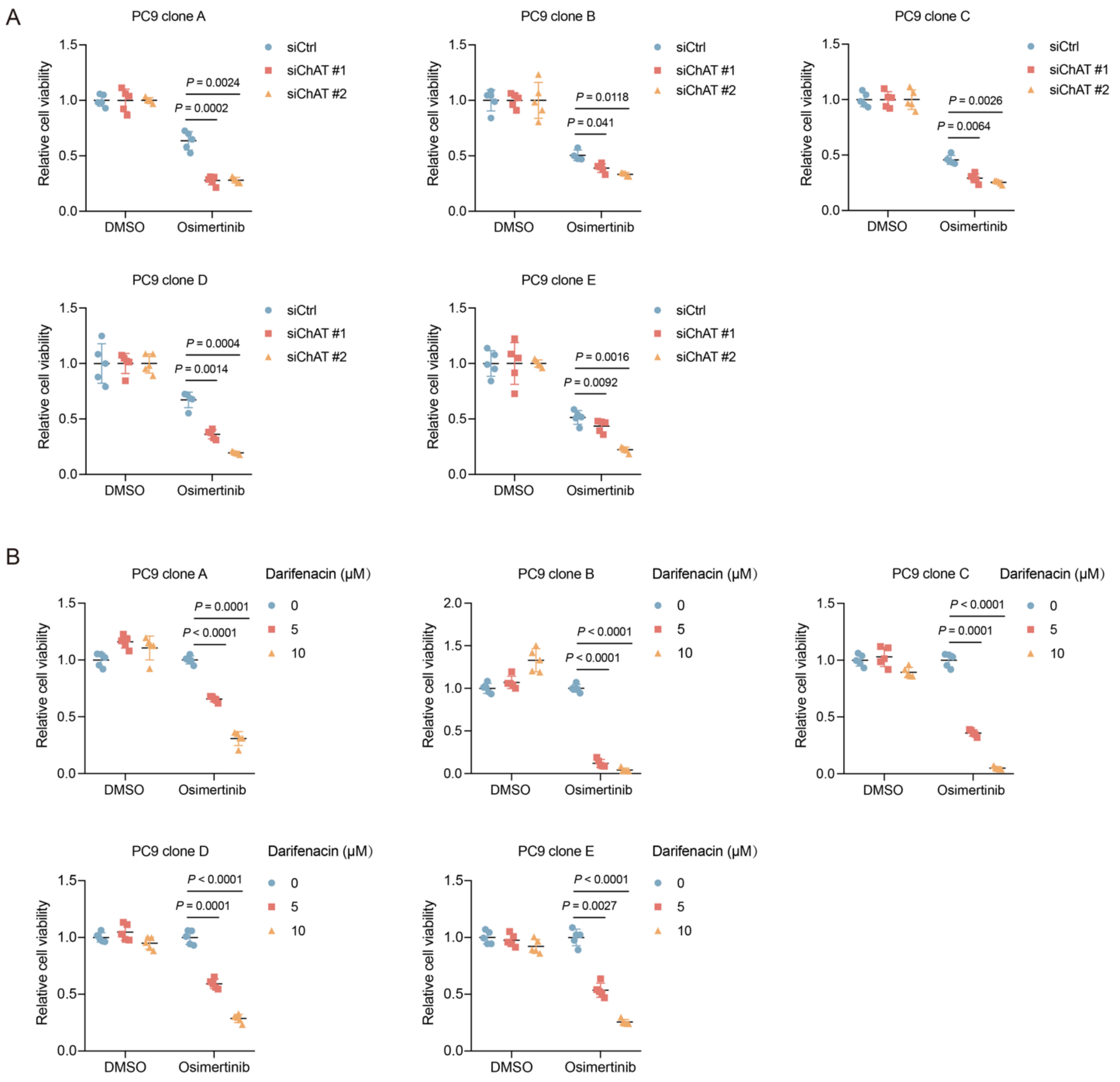

Supplemental Figure 11. The effect of ACh metabolism and signaling inhibition on the drug sensitivity of single cell-derived PC9 clones. (**A**) Relative cell viability of single cell-derived PC9 clones transfected with ChAT or control siRNA for 24 h and followed by indicated concentrations of Osimertinib for 72 h (*n* = 5). (**B**) Relative cell viability of single cell-derived PC9 clones co-treated with indicated concentrations of Osimertinib and Darifenacin for 6 days (*n* = 5). (**A and B**) Data are shown as mean ± s.e.m., two-way ANOVA with Tukey's test was used.

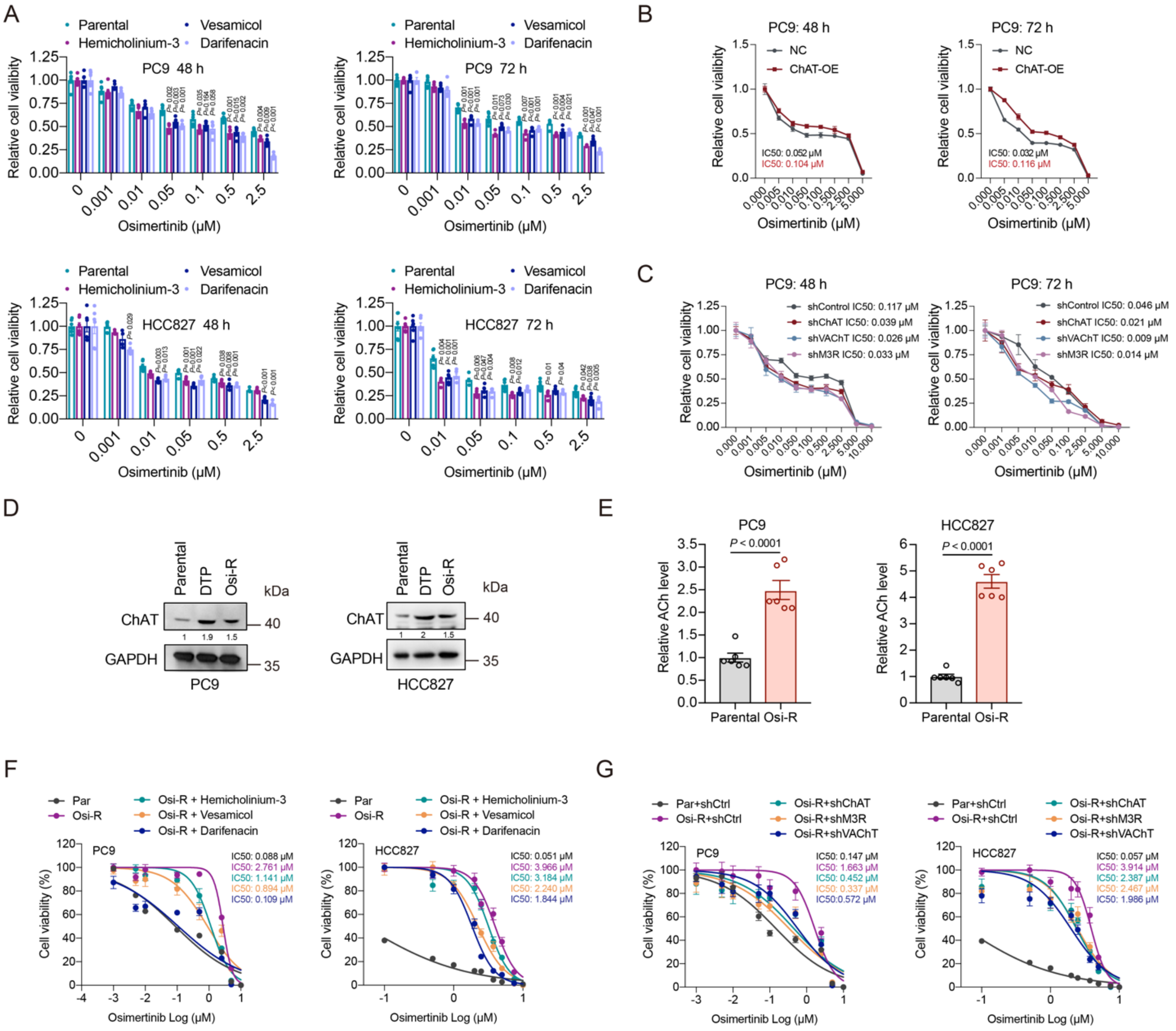

Supplemental Figure 12. The effect of ACh metabolism and signaling inhibition on de novo resistance and acquired resistance. (**A**) Relative cell viability of PC9 and HCC827 cells co-treated with indicated concentrations of Osimertinib and 100 μM Hemicholinium-3, 50 μM Vesamicol and 10 μM Darifenacin for 48 h and 72 h (n = 4-6). (**B**) Relative cell viability of PC9 cells with short ChAT isoform overexpression (ChAT-OE) and negative control (NC) treated with indicated concentrations of Osimertinib for 48 h and 72 h (n = 6). (**C**) Relative cell viability of PC9 cells with shCtrl, shChAT, shVAChT or shM3R treated with indicated concentrations of Osimertinib for 48 h and 72 h (n = 6). (**D**) Western blot analysis of short ChAT isoform in PC9

and HCC827 parental, DTP and Osi-resistant cells. (**E**) Relative abundance of ACh in PC9 and HCC827 parental and Osi-resistant cells (n = 6). (**F**) Dose-response curves to Osimertinib combined with 100 μM Hemicholinium-3, 50 μM Vesamicol and 10 μM Darifenacin in PC9 parental and Osi-resistant cells for 72 h (n = 6). (**G**) Dose-response curves to Osimertinib in PC9 parental and Osi-resistant cells with shCtrl, shChAT, shVAChT or shM3R for 72 h (n = 6). (**A-C** and **E-G**) Data are shown as mean ± s.e.m., two-way ANOVA with Tukey's test (**A**) and two-tailed Student's t test (**E**) was used.

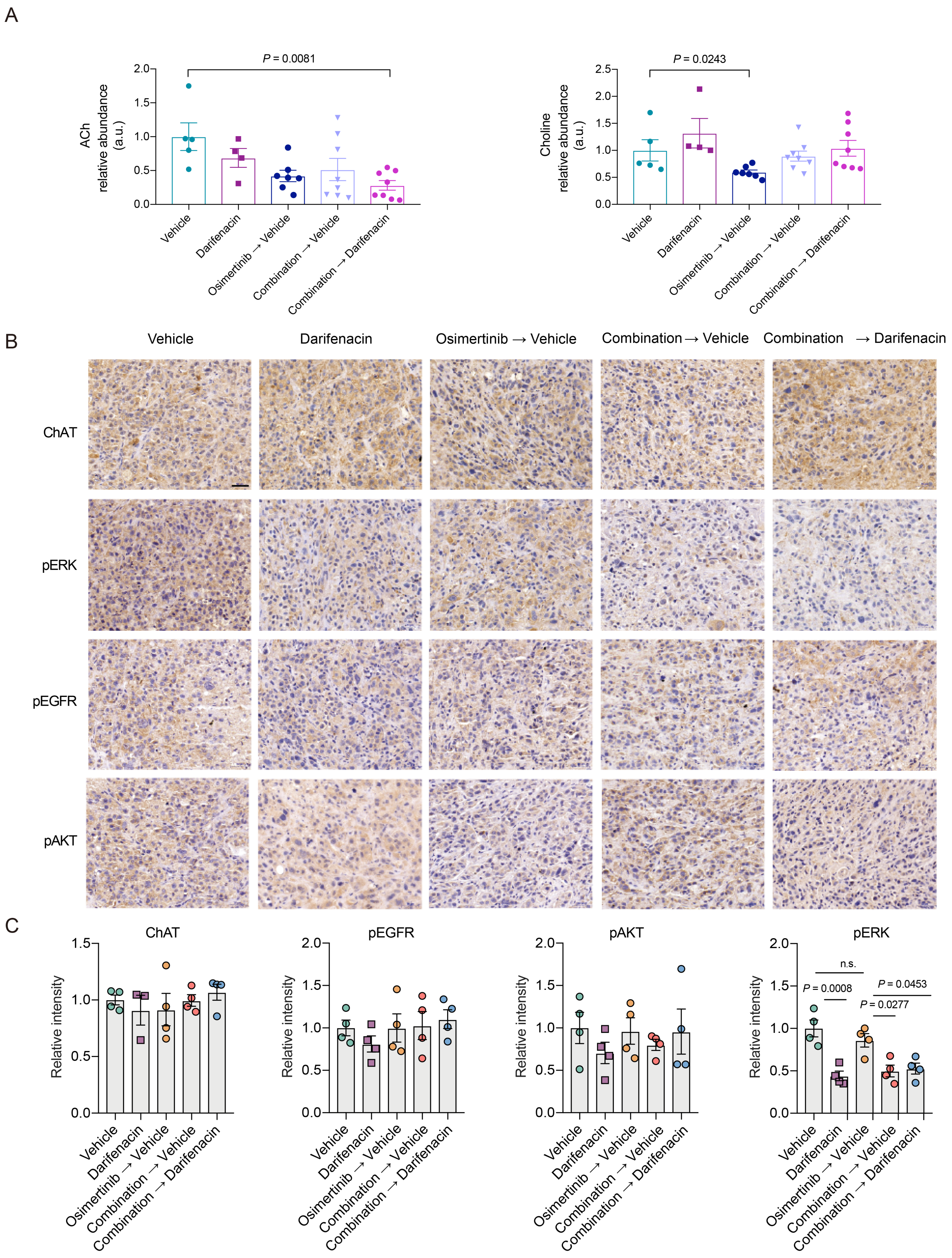

A
P = 0.0081
ACh relative abundance (a.u.)
P = 0.0243
Choline relative abundance (a.u.)
Vehicle
Darifenacin
Osimertinib → Vehicle
Combination → Vehicle
Combination → Darifenacin
B
ChAT
pERK
pEGFR
pAKT
C
ChAT
pEGFR
pAKT
pERK
Relative intensity
n.s.
P = 0.0008
P = 0.0453
P = 0.0277

Supplemental Figure 13. Alterations of the key metabolites and proteins in PC9 xenografts exposed to different treatment strategies. (**A**) Relative abundance of ACh and choline in tumors sections from PC9 xenografts at the end point with pharmacological inhibition. n = 5 mice for Vehicle, n = 4 mice for Darifenacin, n = 7 mice for Osimertinib → Vehicle, n = 8 mice for Combination → Vehicle, n = 8 mice for Combination → Darifenacin. (**B and C**) Representative image and quantification of immunohistochemical staining of ChAT, pERK, pEGFR, pAKT on the tumor sections after indicated treatment. Scale bar, 50 μm. n = 4 mice per group. (**A and C**) Data are shown as mean ± s.e.m., one-way ANOVA with Tukey's test was used.

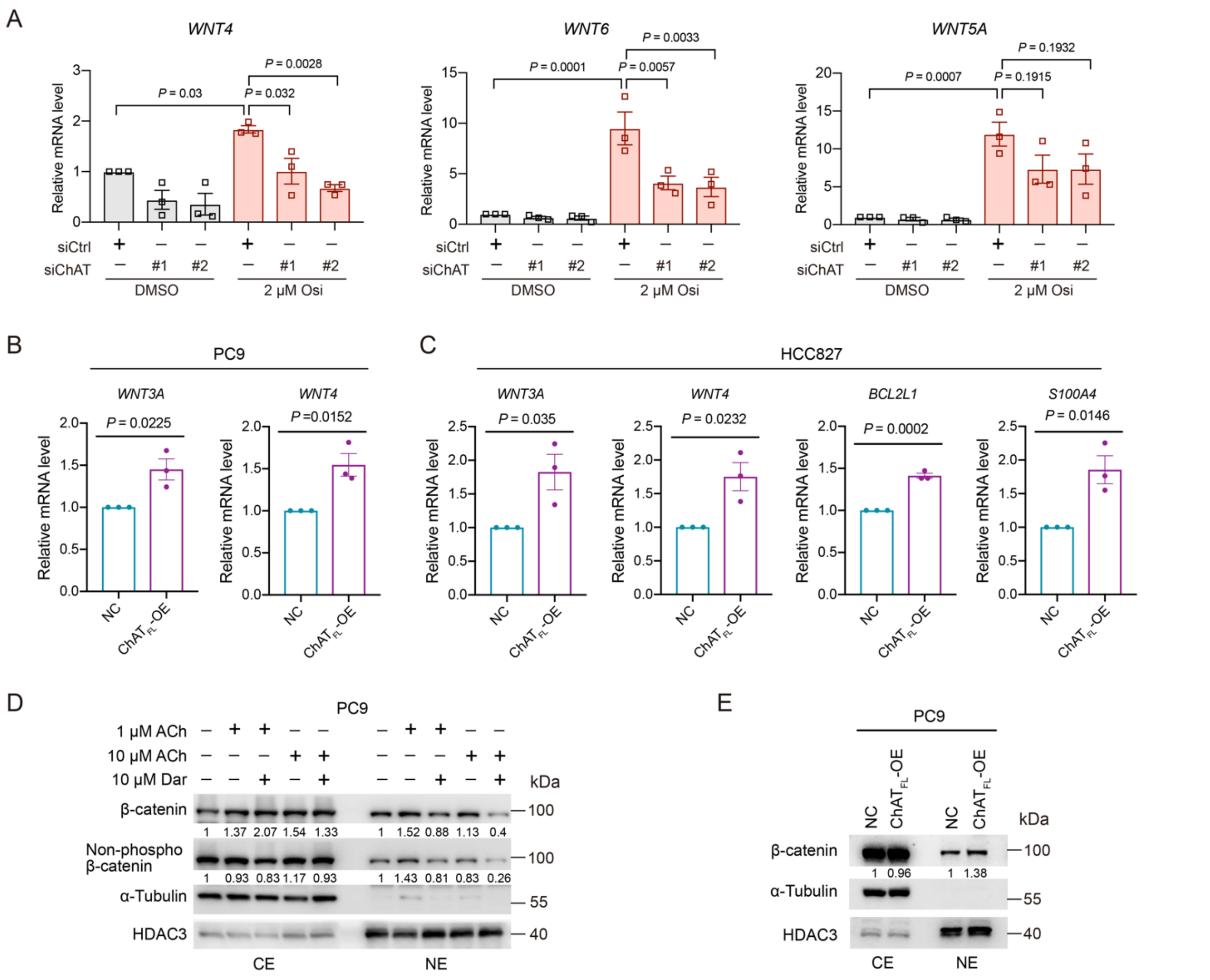

Supplemental Figure 14. The modulatory role of ACh metabolism and signaling on WNT signaling. (**A**) Relative mRNA levels of indicated genes related to WNT signaling in PC9 cells transfected with ChAT or control siRNA and treated with 2 μM Osimertinib for 6 days (n = 3). (**B-C**) Relative mRNA levels of indicated genes related to WNT signaling in PC9 (**B**) and HCC827 (**C**) cells with full-length ChAT overexpression ($ChAT_{FL}$-OE) or negative control (NC) (n = 3). (**D**) Western blot analysis of β-catenin and non-phospho β-catenin in nuclear extract (NE) and cytosol extract (CE) in PC9 cells treated with indicated concentrations of ACh or Darifenacin alone or in combination for 6 days. (**E**) Western blot analysis of β-catenin in nuclear

extract (NE) and cytosol extract (CE) in PC9 cells with $ChAT_{FL}$-OE or NC. (**A-C**) Data are shown as mean ± s.e.m., one-way ANOVA with Tukey's test (**A**) and two-tailed Student's *t* test (**B and C**) were used.

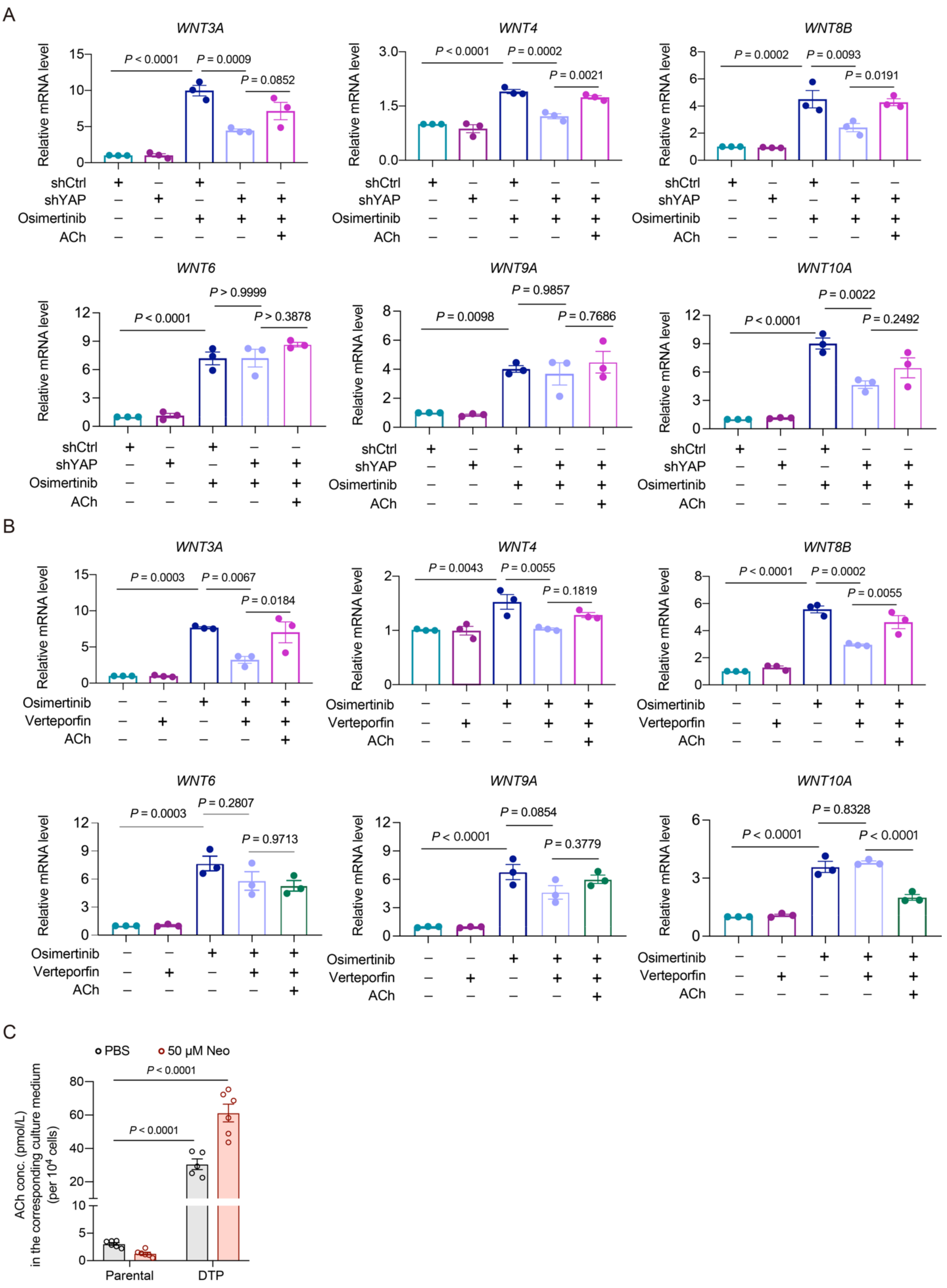
A
WNT3A
WNT4
WNT8B
WNT6
WNT9A
WNT10A
Relative mRNA level
shCtrl
shYAP
Osimertinib
ACh
P < 0.0001
P = 0.0009
P = 0.0852
P = 0.0002
P = 0.0021
P = 0.0093
P = 0.0191
P > 0.9999
P > 0.3878
P = 0.9857
P = 0.0098
P = 0.7686
P = 0.0022
P = 0.2492
B
Verteporfin
P = 0.0003
P = 0.0067
P = 0.0184
P = 0.0043
P = 0.0055
P = 0.1819
P = 0.0002
P = 0.0055
P = 0.2807
P = 0.9713
P = 0.0854
P = 0.3779
P = 0.8328
C
PBS
50 μM Neo
ACh conc. (pmol/L) in the corresponding culture medium (per 10^4 cells)
Parental
DTP

Supplemental Figure 15. The modulatory role of YAP on WNT signaling. (**A**) Relative mRNA levels of indicated genes related to WNT signaling in PC9 cells with shCtrl or shYAP and treated with or without the 2 μM Osimertinib and 10 μM ACh for 6 days (*n* = 3). (**B**) Relative mRNA levels of indicated genes related to WNT signaling in PC9 cells treated with or without 2 μM Osimertinib, 2 μM Verteporfin and 10 μM ACh for 6 days (*n* = 3). (**C**) ACh levels in culture medium of parental PC9 and PC9-derived DTP cells. Neo, Neostigmine, an acetylcholinesterase inhibitor (*n* = 5 or 6). (**A-C**) Data are shown as mean ± s.e.m., one-way ANOVA with Tukey's test (**A and B**) and two-way ANOVA with Tukey's test (**C**) was used.

Supplemental Table 1. Lists of Used GSEA Gene Sets

#cell cycle		#purine metabolism	#pyrimidine metabolism
CDC16	TFDP1	ADCY1	CTPS1
CDC7	ZBTB17	NPR1	DCK
CDC45	CDK1	GUCY2D	DCTPP1
GADD45B	ESPL1	GUCY1A2	DPYD
DBF4	ANAPC10	PDE4C	DTYMK
ANAPC1	RAD21	PDE8B	NME1
CREBBP	BUB1B	PDE5A	PNP
MDM2	ANAPC11	PDE6G	PNPT1
ABL1	RB1	PDE6C	POLA1
SMC1B	SKP2	PDE7B	POLA2
LOC728622	CUL1	ADCY10	POLD2
GADD45G	SMAD3	PDE6A	POLD3
ATM	SMAD4	ADSSL1	POLE2
ATR	ANAPC2	ENTPD8	POLE3
ANAPC7	TFDP2	ADCY9	POLR1B
RBL2	PRKDC	PDE4B	POLR1C
ANAPC5	MAD2L1	GMPR	POLR2D
RBL1	ANAPC4	ENPP1	POLR2G
MYC	SMAD2	NT5E	POLR3G
CDC14B	YWHAQ	ENPP4	POLR3K
SMC1A	CHEK2	PAPSS2	PRIM1
CDC14A	CDC23	PDE9A	PRIM2
LOC731751	EP300	POLR3K	RRM1
SKP1	GSK3B	PRPS2	RRM2
TGFB2	CDKN2A	TWISTNB	TK1
TGFB1	CDKN1C	POLR1C	TWISTNB
GADD45A	CDKN1B	PFAS	TYMS
STAG1	CDKN1A	PPAT	UCK2
LOC650621	CDKN2D	ADSL	UMPS
PLK1	CCNA1	POLD2	UPP1
RBX1	CDKN2B	ADCY3	
STAG2	CDKN2C	POLE3	
TGFB3	FZR1	POLA2	
MCM4	SMC3	AK2	
ORC6	ANAPC13	PRIM2	
CCND1	PCNA	POLR2D	
MAD1L1	TTK	POLR1B	
MCM3	PKMYT1	DCK	
MCM6	CDK2	POLD3	
MCM5	CDC26	GDA	
YWHAB	E2F5	GART	
CCNA2	CDK4	GMPS	
MCM7	WEE1	POLA1	
BUB1	E2F4	POLR2G	
CHEK1	E2F3	PAICS	
WEE2	TP53	PNPT1	
MCM2	E2F2	RRM1	
PTTG1	ORC1	AK5	
CDC27	ORC2	XDH	
CDC25B	CCNE2	AK6	
CDC25C	CDK6	HPRT1	

LOC651610	ORC4	PRIM1	
CDC25A	CCNB2	NME1	
CDC6	CDK7	POLE2	
CDC20	MAD2L2	PNP	
BUB3	ORC5	POLR3G	
YWHAZ	HDAC1	RRM2	
CCND2	HDAC2	PDE11A	
YWHAH	CCNH	PDE2A	
CCNB1	CCNB3	AK4	
YWHAG			
YWHAE			
CCNE1			
ORC3			
CCND3			
PTTG2			
SFN			
E2F1			

Supplemental Table 2. Clinical information of NSCLC patients for plasma sample collection

Patient ID	stage	EGFR mutation	EGFR TKI	Response rate	PFS	PFS status
P01	T4N2M1B-IV	19del	Osimertinib	PR	4.5	1
P02	T4N2M1B-IV	L858R	Osimertinib	SD	4.2	1
P03	T4N1M1A-IV	19DEL+T790M	Osimertinib	PR	14	1
P04	T4N2M1B-IV	19del	Osimertinib	PR	10.4	1
P05	T4N2M1A-IV	L858R	Osimertinib	PR	7	1
P06	T4N3M1B-IV	L858R	Osimertinib	PR	21.5	0
P07	T4N2M1B-IV	L858R+T790M	Osimertinib	PR	9.9	1
P08	T4N3M1B-IV	19del	Osimertinib	SD	32.4	1
P09	T1AN3MO-IIIB	19del	Osimertinib	PR	5.6	0
P10	T3N3M1B-IV	L858R	Gefitinib	PD	1.2	1
P11	cT3N2M1c-IVB	19del	Icotinib	PR	17.4	0
P12	cT4N3M1c-IVB	L858R	Gefitinib	PR	9.9	1
P13	cT2bN3M1c-IVB	19del	Gefitinib	PR	18.5	0
P14	cT4N3M1c-IVB	19del	Icotinib	PR	20.5	1
P15	cT2aN0M1b-IVA	19del	Gefitinib	PR	10	0
P16	cT2N1M1a-IVA	L858R	Gefitinib	PR	16.1	1
P17	cT1cN2M1c-IVB	19del	Gefitinib	PR	17.4	0
P18	cT4N3M1c-IVB	L858R	Gefitinib	PR	6.1	1
P19	cT1cN3M0-IIIB	19del	Icotinib	PR	18.6	1
P20	cT4N3M1c-IVB	L858R	Gefitinib	PR	11	0
P21	cT3N3M1a-IVA	19del	Icotinib	PR	14.3	1
P22	cT2aN3M1a-IVA	L858R	Icotinib	PR	8.3	1
P23	cT4NxM1a-IVA	19del	Icotinib	PR	16	0
P24	T4N3M1a-IVA	L858R	Gefitinib	PR	9	1
P25	cT1bN3M1a-IVA	19del	Icotinib	PD	1.3	1
P26	cT2aN2M1b-IVA	19del	Gefitinib	PR	8.5	0
P27	cT4N2M1c-IVB	19del	Gefitinib	PR	14.2	1
P28	cT1cN3M0-IIIB	19del	Gefitinib	PR	9.1	0
P29	cT3N3M1b-IVA	19del	Icotinib	PR	18	0
P30	cT4N2M0-IIIB	19del	Gefitinib	PR	9.3	0
P31	cTxN3M1c-IVB	L858R	Icotinib	PR	6.3	1
P32	cT4N3M1c-IVB	G719X	Afatinib	SD	2.1	1
P33	IVB	L858R	Icotinib	SD	12.7	1
P34	IVA	19del	Gefitinib	PR	13.9	1
P35	IVA	19del+S768I	Afatinib	PR	6.6	1
P36	IVB	L858R	Icotinib	SD	6.6	1
P37	IIIB	19del	Afatinib	PR	7.5	1
P38	IVA	19del	Icotinib	PR	8.9	1
P39	IIIB	19del	Icotinib	PR	30.4	1
P40	IVB	19del	Gefitinib	PR	7.3	1
P41	IIIB	L858R	Gefitinib	PD	1.5	1
P42	IVB	L858R	Gefitinib	SD	20	1
P43	IVA		Icotinib	SD	11.7	1
P44	IVA	L858R	Gefitinib	SD	28.2	1
P45	IVA	19del	Gefitinib	PR	2.8	1
P46	IVB	L858R	Icotinib	SD	4.4	1
P47	IVB	19del	Gefitinib	PR	30	1
P48	IVB	L858R	Erlotinib	PD	3.9	1
P49	IVA	19del	Gefitinib	PR	12	1
P50	IVB	19del	Gefitinib	SD	5.4	1
P51	IVB	L858R	Gefitinib	PR	4	1

P52	IIIC	L858R	Gefitinib	PR	16.1	1
P53	IB	L858R	Icotinib	PR	23.5	1
P54	IVB	19del	Gefitinib	SD	4.6	1
P55	IVA	19del	Gefitinib	PR	10.9	1
P56	IVA	19del	Icotinib	PR	25.9	1
P57	IVA	19del	Gefitinib	PR	21.3	1
P58	IVB		Gefitinib	PR	19.7	1
P59	IVA	L858R	Erlotinib	PR	6.3	1
P60	IVB	19del	Icotinib	SD	12.8	1
P61	IVB	L858R	Gefitinib	SD	5.7	1
P62	IVA	19del	Gefitinib	SD	4	1
P63	IVB	L858R	Gefitinib	SD	6.5	1
P64	IIIB	L858R	Gefitinib	PR	28.5	1
P65	IVA	L858R	Icotinib	PR	13.8	1
P66	IVA	19del	Icotinib	PR	22.4	1
P67	IVA	19del	Gefitinib	PR	13.2	1
P68	IVB	L858R	Gefitinib	SD	7.2	1
P69	IVB	L858R	Gefitinib	PR	17.2	1
P70	IVA	L861Q	Afatinib	SD	28.6	1
P71	IVB	19del	Gefitinib	PR	26.9	1
P72	IVA	19del	Gefitinib	SD	10.3	1
P73	IVA	19del	Gefitinib	PR	18.5	1
P74	IIIC	L858R	Gefitinib	SD	6.1	1
P75	IVA		Erlotinib	SD	12.6	1
P76	IVA	L858R	Gefitinib	PR	20.8	1
P77	IVB	19del	Gefitinib	SD	11.5	1
P78	IVB	L858R	Erlotinib	PR	9.1	1

Supplemental Table 3. Clinical information of NSCLC patients for paired tumor tissue

Patient ID	Stage	EGFR mutation	EGFR TKI	Response rate
T01	IIIC	L861Q	afatinib	PR
T02	IVB	L858R	gefitinib	PD
T03	IVB	19DEL	afatinib	PR
T04	IVA	L858R	icotinib	SD
T05	IVB	L858R	gefitinib	SD
T06	IIIA	19DEL	icotinib	SD
T07	IVB	L858R	gefitinib	PR
T08	IVA	19DEL	gefitinib	PR
T09	IVB	L858R	gefitinib	PR
T10	IVB	19DEL	erlotinib	PR
T11	IVA	L858R	gefitinib	PR
T12	IIIB	19DEL	gefitinib	PR
T13	IVA	19DEL	erlotinib	PR
T14	IVB	19DEL	gefitinib	PR
T15	IVA	19DEL	gefitinib	PR
T16	IVB	19DEL	gefitinib	PR
T17	IVA	19DEL+L861Q	gefitinib	PR
T18	IVB	L858R	icotinib	PD
T19	IVB	G719X	afatinib	PR
T20	IVB	L858R	gefitinib	PR

Supplemental Table 4. Small molecule compound screen

Category	Parameter	Description
**Assay**	Type of assay	ATP- measurement in vitro
	Target	mAChR and/or nAChR
	Primary measurement	luminescent detection of ATP
	Key reagents	CellTiter-Glo Luminescent Cell Viability Assay kit (Promega), osimertinib (Selleck), rociletinib (Selleck), erlotinib (Selleck) and small molecule libraries.
	Assay protocol	Detailed method is described in the 'Drug Screen' section in Methods.
**Library**	**Library size**	**26 compounds**
	Library composition	Darifenacin HBr: Selleck (S3144)
		Ipratropium Bromide: Selleck (S1683)
		Oxybutynin: Selleck (S1754)
		Irsogladine: Selleck (S1929)
		Methscopolamine: Selleck (S1978)
		Atropine sulfate monohydrate: Selleck (S2130)
		Scopolamine HBr: Selleck (S2508)
		Pancuronium dibromide: Selleck (S2497)
		Gallamine triethiodide: Sigma-Aldrich (G8134)
		DL-Homatropine hydrobromide: Sigma-Aldrich (H0126)
		p-Fluorohexahydro-sila-difenidol hydrochloride: Sigma-Aldrich (H127)
		Hexamethonium bromide: Sigma-Aldrich (H0879)
		MG624: Sigma-Aldrich (M3184)
		Mecamylamine hydrochloride: Sigma-Aldrich (M9020)
		Methoctramine hydrate: Sigma-Aldrich (M105)
		Orphenadrine hydrochloride: Sigma-Aldrich (O3752)
		Pirenzepine dihydrochloride: Sigma-Aldrich (P7412)
		Propantheline bromide: Sigma-Aldrich (P8891)
		Succinylcholine chloride dihydrate: Sigma-Aldrich (S8251)
		DL-Trihexyphenidyl hydrochloride: Sigma-Aldrich (T1516)
		Tetraethylammonium chloride: Sigma-Aldrich (T2265)
		Telenzepine dihydrochloride hydrate: Sigma-Aldrich (T122)
		Tropicamide: Sigma-Aldrich (T9778)
		Benztropine mesylate: Sigma-Aldrich (B8262)
		Biperiden hydrochloride: Sigma-Aldrich (B5311)
		4-DAMP methiodide: Sigma-Aldrich (D104)
	Source	Original sources are listed above.
**Screen**	**Format**	**384 well**
	Concentration(s) tested	1.25, 2.5, 5, 10, 20, 50 μM compound, < 0.5% DMSO
	Plate controls	DMSO-containing (as parental cell group) and 2 μM EGFR TKI-containing medium (as DTP cell group) represented 100% cell viability (0% inhibition)
	Reagent/ compound dispensing system	Labcyte-Echo 550
	Detection instrument and software	BioTek Cytation3
	Normalization	The relative survival of parental PC9 cells and EGFR TKI-induced tolerant PC9 cells in the presence of compounds was normalized against respectively negative control conditions. The wells with DMSO or 2 μM EGFR TKI were averaged to determine the average signal without a compound. The signal for each compound on each

		plate was divided by the average negative control signal to give a % cell viability value. [(signal with compound)/(average signal with negative control)] x 100% = % cell viability.
